# Advances in Troubleshooting Fish and Seafood Authentication by Inorganic Elemental Composition

**DOI:** 10.3390/foods10020270

**Published:** 2021-01-29

**Authors:** Maria Olga Varrà, Sergio Ghidini, Lenka Husáková, Adriana Ianieri, Emanuela Zanardi

**Affiliations:** 1 Department of Food and Drug, University of Parma, Strada del Taglio 10, 43126 Parma, Italy; mariaolga.varra@studenti.unipr.it (M.O.V.); sergio.ghidini@unipr.it (S.G.); adriana.ianieri@unipr.it (A.I.); 2 Department of Analytical Chemistry, Faculty of Chemical Technology, University of Pardubice, Studentska 573 HB/D, CZ-532 10 Pardubice, Czech Republic; lenka.husakova@upce.cz

**Keywords:** fraud, authentication, traceability, fish, geographical origin, multi-elemental profile, stable isotopes, ICP-MS, ICP-OES, chemometrics

## Abstract

The demand for fish and seafood is growing worldwide. Meanwhile, problems related to the integrity and safety of the fishery sector are increasing, leading legislators, producers, and consumers to search for ways to effectively protect themselves from fraud and health hazards related to fish consumption. What is urgently required now is the availability of reliable, truthful, and reproducible methods assuring the correspondence between the real nature of the product and label declarations accompanying the same product during its market life. The evaluation of the inorganic composition of fish and seafood appears to be one of the most promising strategies to be exploited in the near future to assist routine and official monitoring operations along the supply chain. The present review article focuses on exploring the latest scientific achievements of using the multi-elemental composition of fish and seafood as an imprint of their authenticity and traceability, especially with regards to the geographical origin. The scientific literature of the last 10 years focusing on the analytical determination and statistical elaboration of elemental data (alone or in combination with methodologies targeting other compounds) to verify the identity of fishery products is summarized and discussed.

## 1. Introduction

The verification of food authenticity and integrity is a complex topic which has become a matter of public interest in recent years. This issue involves many different aspects, from the identification of mislabeling and misrepresentation to adulteration and contamination of the product.

Today, the traceability of fish and seafood and detection of intentional and unintentional fraud is a challenging task, as the supply chain of fishery products is among the most diversified and globalized. As a matter of fact, fish is currently among the most frequently misdescribed foodstuffs worldwide, to a point that almost 20% of fish in the sail and restaurant sectors of 55 countries has been recently found to be misdescribed [1]. Specifically, the major economic losses affecting the sector derive from the substitution of highly valuable fish and seafood species for morphologically similar but lower-quality ones and from the increasingly common falsification in relation to the geographical origin. Albeit these fraudulent practices seem to have a negative impact only from an economical point of view, some health implications may arise, for example, from the replacement of certain fish species with cheaper but potentially poisonous ones [2], or from the sale of illegally caught fish originating from polluted areas [3].

In order to prevent fraud, protect producers and consumers, and promote high-quality fish products, the reinforcement of the international food monitoring program is not sufficient. Indeed, control measures are required to be undertaken in synergy with the implementation of proper vulnerability assessment systems and the development of rapid analytical tools, so as to confidently verify whether a product is genuine or counterfeit and to guarantee the integrity of the whole production chain.

Many different biological and chemical methods have been developed over the years to ascertain the authentic nature of a wide range of foodstuffs. These methods focus on the evaluation of the organic (DNA, proteins, lipids, sugars, and/or metabolites) and inorganic (elements, isotope ratios) fractions of food and exploit the principles of chromatography, mass spectrometry, and spectroscopy to identify, in a targeted way, few or multiple compounds acting as secondary markers of authenticity [4]. In this sense, the determination of the inorganic multi-elemental signatures (in terms of major, trace, and ultra-trace elements), accompanied by multivariate statistics, is increasingly applied to authenticate different foods of animal origin such as honey [5], pork meat [6], and cheese [7], especially in relation to the geographical origin and the method of production. In this context, the evaluation of the elemental content of fish and seafood is particularly advantageous, since it may allow for the simultaneously monitoring of mislabeling and of the maximum acceptable regulation limits for certain toxic elements established at the European level [8], thus pursuing both integrity and safety objectives.

Elements found in fish tissue are scientifically recognized to be a reflection of the elemental composition of the overall surrounding environment, from the aquatic habitat to the production premises, and this is particularly advantageous when the country of origin of wild specimens is thought to be identified. On the contrary, the elemental content of farmed specimens is inevitably affected by feeding stuffs, both from a qualitative and a quantitative point of view. Thus, using the elemental profile of the tissues of farmed specimens to trace back to the country of origin may be problematic by virtue of the fact that the same feed can be traded internationally and given to fish cultured in different parts of the world.

When working with the element composition of fish for authentication purposes, it should be taken into consideration that the presence of elements in the aquatic environment explored by the fish during life is not only dependent on the specific geochemical characteristics of the habitat, but it may be significantly influenced by other environmental factors either of natural (such as climate, water temperature, salinity, age, and sexual maturity of the animal) or anthropic (i.e., the exogenous pollution) origin [9]. In addition, after catch, fishery products are more frequently handled and enlivened compared to other foodstuffs. Therefore, the likelihood of unwanted and misleading elements being incorporated as contaminants throughout the whole production cycle increases significantly.

For these reasons, the overall elemental signature needs to be strictly evaluated before being used as a tool to address authenticity problems of fish and seafood. In this regard, chemometrics and machine learning have now become an essential support for increasing the strength and reliability of high-throughput analytical techniques. As a matter of fact, the advanced statistical elaboration of elemental data has already been proven to be a straightforward and effective means to study elements’ behavior; identify common but hidden compositional characteristics among similar food samples; separate complementary, opposite, or redundant information enclosed into elemental data; define classification rules; and simplify the overall methodology by extracting the effectively significant elemental markers for classification [10].

The present review article was aimed at discussing the applications and advances in data mining of the multi-elemental profile of fish, mollusks, echinoderms, and crustaceans from the last 10 years as a strategy to verify whether mandatory labelling information matches the identity of these products. The survey took into consideration the elemental measurements performed only on edible tissues of fishery products, which, albeit being more rapidly subjected to variations induced by environment compared to hard structures such as otoliths, statoliths, skeleton, and scales, are retained in the final traded products and hence potentially monitorable in every phase of the production chain. As evidenced below, only raw products were discussed, since, as far as we know, no considerable breakthroughs in tracing and authenticating transformed (e.g., salted, smoked, marinated) fish and seafood products have been achieved.

## 2. Analytical and Chemometric Methodologies for Element and Stable-Isotope Analysis of Fish and Seafood

Various analytical techniques have been used for the determination of the elemental content of fish and seafood throughout the last years [9,11,12,13]. Among these, atomic spectroscopic methods, such as atomic absorption spectrometry (AAS) [14,15,16,17,18,19,20] with flame [16,19,20] or electrothermal atomization [16,17,18], atomic fluorescence spectrometry (AFS) [21] inductively coupled optical emission spectrometry (ICP-OES) [16,22], inductively coupled plasma mass spectrometry (ICP-MS) [23,24], and X-ray fluorescence (XRF) [19,25], have been the most frequently employed. On the contrary, electroanalytical [26] or neutron-activation-based techniques, such as neutron activation analysis (NAA), have been used to a lesser extent [27,28,29].

These techniques offer specific advantages and, at the same time, present some limitations which make their application preferable in some cases but not in others. The main characteristics and performances of the analytical methods that can be used for comparing the multi-element or stable-isotope composition of fish and seafood samples are examined in the text below, while a comprehensive and detailed overview of the main benefits and drawbacks is outlined in the Supplementary File (Table S1).

In the case of major and some minor elements, AAS and OES with flames (flame atomic absorption spectroscopy, FAAS, and flame optical emission spectroscopy, FOES), are still valuable and well-established techniques that are routinely and customarily applied in the area of fish and seafood analysis due to their robustness in relation to interferences and sample introduction problems, selectivity, straightforward application, and lower cost [16,19,20,30]. On the other hand, these methods still present limitations related to sensitivity. Thus, electrothermal AAS (ET-AAS) [16,17,28], hydride generation AAS (HG-AAS) [31], and cold vapor AAS (CV-AAS) [15,28] or direct thermal decomposition AAS [19,32] are employed in the lower concentration range. However, the main disadvantage is that AAS is primarily limited to the determination of metallic elements and is a single-element technique with a linear range typically less than two orders of magnitude. Despite being used in only in a very limited number of cases [18], high-resolution continuum source AAS (HR-CS-AAS) is overcoming some of the limitations of AAS, as it allows for the simultaneous evaluation of several absorption lines in the selected spectral range, accurate background correction, and the determination of nonmetals.

ICP-OES is by far the most commonly applied technique for the analysis of food samples [16,18,22,33] because it offers simultaneous multi-element measurement, capabilities for sensitive determination of refractory elements, quantification of nonmetals, and high analytical throughput. Microwave induced plasma optical emission spectrometry (MIP-OES) using the magnetically excited microwave plasma source has also been recently applied to fish and seafood [34,35], mainly because it is characterized by detection limits down to sub-ppb levels, significant cost reduction, and simpler spectra than ICP-OES. However, at present, both ICP-OES and MIP-OES fail to meet the needs required in routine applications when the determination of elements at trace or ultra-trace concentrations is in demand.

ICP-MS is better suited to meet this task and is currently a frontline technology, rapidly replacing other methods in many fields of food science. Unsurpassed advantages, such as high sensitivity, selectivity, wide dynamic concentration range up to 11 orders of magnitude, high sample throughput, and multi-analyte capabilities, make this method an ideal candidate for food authentication studies, since it might facilitate the discrimination and classification of samples [23,36,37].

More detailed technical aspects of the abovementioned methodologies can be retrieved from literature [37,38].

The analysis of biologic matrices such as foodstuffs by atomic and mass spectrometry methods, especially at trace and ultra-trace levels, is often a difficult and challenging task. As a matter of fact, a quite complex biological matrix poses problems related not only to sample heterogeneity, the selection of proper sample treatment, and decomposition, but also matrix interferences.

In the ICP-MS analysis of fish and seafood samples, both spectral and non-spectral interferences are expected to be encountered [18,23,39]. Whereas non-spectral effects can be easily overcome using a proper calibration strategy, including the use of an internal standard [18,23], standard additions, and/or the isotope dilution, spectral effects due to the overlaps by different polyatomic ions (formed from the combination of species derived from the matrix elements, plasma gas and sample solvents) are more serious and difficult to handle [40].

High-resolution mass spectrometers with a sector field mass analyzer could be the ideal solution to bypass most of these problems [40]. However, owing to their high price, these instruments are not easily accessible for most laboratories.

Time-of-flight (TOF)-ICP-MS instruments have several advantages, such as fast simultaneous multi-elemental analysis, improved precision of measurements of the isotope ratios, very low volume of the sample needed for the analysis, and tolerance to higher salinity of samples. However, they do not have the adequate resolution to eliminate the spectral interferences typically encountered when analyzing biological samples. As a result, mathematical corrections must be employed, but this approach is less effective when performing trace analysis [41]. The absence for effective solutions related to the control of problematic spectral effects, which were not accessible to users until recently, has limited the widespread diffusion of this technique in routine practice. Nevertheless, there is an increasing trend in resorting to the use of collision cell technology for interference management during sample analysis in the current TOF-ICP-MS instrumentation [42]. At present, the quadrupole-based ICP-MS equipped with a collision/reaction cell (CRC) for the elimination of spectral interferences is the most popular ICP-MS instrumentation on the market. In the reaction cell mode, interfering ions are removed by the transformation into different species or uncharged atoms or molecules through specific chemical reactions with a supplementary reaction gas (H_2_, NH_3_, O_2_, N_2_O, or CH_4_) [40]. Although this approach is more efficient for the removal of known spectral interferences, it may lead to a formation of new unwanted interfering polyatomic ions. The collision cell mode is instead more suitable for the multi-elemental analysis of unknown samples. For this purpose, He is widely used as a collision gas to slow down polyatomic interfering ions to a larger extent than the atomic analyte ions, such that the former could be selectively discriminated against on the basis of their lower kinetic energy.

With the introduction of an ICP-tandem mass spectrometer (MS/MS, often referred to as triple quadrupole ICP-MS or ICP-QQQ), the CRC technology in quadrupole-based ICP-MS has greatly improved [43]. This instrumentation, equipped with CRC located between two quadrupole mass filters, provides an elegant approach via a precursor ion and/or product ion scanning to solve even the most challenging cases of spectral overlap and interference. Moreover, it can determine a wider range of analytes at much lower concentrations with greater reliability and higher confidence [43].

In addition to total element determinations, the current ICP-MS instrumentation is suited also to isotope ratio analysis, even if the isotope ratio precision is strictly dependent on the type and the design of the instrument used. Considering that the simultaneous measurement of multiple isotopes provides a better precision in isotope ratio measurement, the use of TOF-ICP-MS or multi-collector mass spectrometer with a plasma source for ionization (MC-ICP-MS) is considerably more advantageous than the use of a single quadrupole ICP-MS for isotope analysis. However, the commonly used mass spectrometers typically do not provide the sensitivity and precision required for the determination of light isotopes ratios. In addition, they are susceptible to isotopic fractionation (mass bias). Therefore, isotope ratio mass spectrometry (IRMS) [44,45], nuclear magnetic resonance (NMR) [46,47], and thermal ionization mass spectrometry (TIMS) [12] are more suitable for this purpose.

Atomic fluorescence spectrometry (AFS) may represent an alternative to the other atomic and mass spectrometric techniques, as it provides low detection limits, wide linear calibration range, simplicity, and lower acquisition and running costs. These analytical features make AFS superior to AAS and equal to ICP-MS or ICP-OES [22,48], especially in speciation studies, as long as single element speciation studies are considered [36].

Recently, there has been an increase in the application of nondestructive multielement methods for analysis of seafood samples [25,49]. Methods based on X-ray spectrometry such as X-ray fluorescence (XRF) [19,25,49], energy dispersion-XRF (ED-XRF) [19], proton induced X-ray emission (PIXE), total reflection X-ray fluorescence spectrometry (TXRF), and synchrotron X-ray fluorescence (SXRF), as well as methods based on X-ray microanalysis, offer several benefits [50]. Among these, the selective detection and sensitivity (about mg kg^−1^ and below) for most of the elements [12,49], minimal sample preparation, high sample throughputs, and accuracy in quantification are worth mentioning [50]. In addition, field portable-XRF analyzers are becoming increasingly popular for a wide variety of elemental analysis applications [50].

Laser-based techniques also play an important role for the direct analysis of solid samples and, in the last years, they have become increasingly present in the food industry. Laser-induced breakdown spectrometry (LIBS) is considered a promising micro-destructive food analysis tool for rapid qualitative and quantitative chemical analysis [50,51]. However, the direct analysis of samples with complex organic matrices such as fresh food products is not easy [50]. As a matter of fact, it is often not possible to analyze the sample without any preparation, since the results might be misleadingly affected by any inhomogeneity of the material. On the other hand, the sample preparation for LIBS analysis is minimal when compared to reference methods such as AAS or ICP-MS. The major limitation of LIBS for practical applications results from its reduced sensitivity for minor mineral elements and heavy metals, with very low concentrations in a complex organic matrix.

The connection of laser ablation (LA) with ICP-MS [52,53,54] represents a quite versatile analytical tool, offering the fastest analytical speed compared to all the other techniques, favorable limits of detection (approaching ppb levels), capability for performing bulk analysis, depth profiling, and elemental/isotope mapping [12]. Nevertheless, LA-ICP-MS still lacks sufficiently matrix-matched reference materials for each considered matrix type, and the analysis accuracy is restricted by several factors, such as sensitivity drift, elemental/isotopic fractionation, and matrix effects [50,55].

Electrothermal vaporization (ETV) is also an efficient and powerful approach for a bulk analysis where solid samples can be directly turned into aerosols [50,55]. This strategy significantly boosts ICP-MS quantitative applications in desired field [56].

### 2.1. Sample Digestion Procedures for Elemental Analysis

The market of most of the abovementioned analytical apparatus, such as AAS, AFS, and those which make use of a plasma source for ionization, offers mainly instrumentation dedicated to the analysis of liquid samples. Consequently, digestion procedures for solid samples are necessarily required. Furthermore, sample preparation is a crucial issue for food products due to their inhomogeneity and matrix complexity.

Nowadays, the most used and useful digestion technique for a wide range of analytes and sample matrices is the high-pressure digestion using a closed-vessel microwave system [15,16,17,18,20,24,34,36,41,49,57]. This technique increases the sample throughput, minimizes analyte losses during the decomposition, reduces both contamination risk (especially for trace analytes) and consumption of reagents, and is more effective, resulting in low residual carbon content of digested samples [57]. In addition to high-pressure closed-vessel microwave digestion, digestion involving opened vessels or classical dry-ashing digestion is generally performed. In wet-acid digestion, HNO_3_ alone [15,16,20,24] or combined with H_2_O_2_ [17,18,28,34,36,41,49] and, occasionally, HCl [35] or HClO_4_ [22,23,33], is the most commonly used reagent. However, several novel approaches or adaptations to established procedures for sample preparation have been recently introduced. In particular, a growing interest toward the use of diluted and nonhazardous analytical reagents is now emerging, in accordance with green chemistry and the need to reduce the negative impact of chemical analyses on the environment [24,57]. From this standpoint, ultrasound-assisted extraction and microwave-assisted extraction [31,36,57] seem to be very promising approaches for sample preparation in the near future, allowing for the optimization of working times and consumption of analytical reagents.

### 2.2. Multivariate Data Analysis and Machine Learning

The growing interest in high-throughput element-based methods to characterize foodstuffs may be partly justified by the efforts in the field of multivariate data analysis and machine learning, which have significantly simplified data handling and improved the identification of food fraud. Multivariate qualitative methods are well established in the field of analytical chemistry oriented toward the authenticity and adulteration verification of foodstuffs, and the development of new algorithms for classification is continuously increasing [10]. Despite this, analysis of the literature revealed that the statistical analysis of the multi-elemental profile of fish has been mostly limited to the classical use of principal component analysis (PCA) and cluster analysis (CA) as exploratory (unsupervised) tools. As for sample classification purposes, hard modelling of data based on linear discriminant analysis (LDA) and canonical discriminant analysis (CDA) has been more frequently employed (see Table 1). This is probably due to the fact that the theoretical background of these data elaboration techniques is more consolidated among the scientific community compared to other more modern hard-modelling discriminant techniques such as partial least square discriminant analysis (PLS-DA) and soft-modelling techniques such as soft independent modelling of class-analogy (SIMCA) [58]. In addition, the applied methodologies appear to lack of proper validation protocols to be followed, which are necessary for the development of reliable and transferable multivariate-based models for foods classification [59].

Various techniques, including K-nearest neighbors (KNN), K-mean clustering, and artificial neural network (ANN), are crucial for future successful development of prediction models to food authentication solutions.

Further details on chemometrics and machine learning techniques applied to food science can be found in the literature [10,60].

## 3. Authentic Elemental Signature of Fish and Seafood

As discussed below, authentication and traceability studies have often been performed by coupling elemental analysis (major, trace, and/or ultra-trace elements) with other techniques targeting other compounds, with the objective to increase the specificity of discrimination and obtain better results.

The merging of data from the isotopic analysis and elemental analysis of light (H, C, N, O, and S) and heavy elements (Sr, Pb) has been the most frequently investigated analytical strategy to approach traceability problems of fish and seafood.

The rationale behind this research trend over the years lies in the strong correlation between any variation in isotope fractionation (ratio between isotopes of a specific elements) and the geological, pedological, and wheatear characteristics of a given geographical area [71]. Among these, the isotopic distribution of light elements such as O (δ^18^O, ^18^O/^16^O), and H (δ^1^H, ^1^H/^2^H) in fishery products is influenced by the original isotopic distribution of the same elements in the water basin from which the fish come from, which, in turn, is the reflection of the isotopic distribution in the rainfall of the specific area [72]. More, the isotope ratio of C (δ^13^C, ^13^C/^12^C) in fish tissues may be related to the type of vegetation eaten by the fish during its life. In particular, the plants are characterized by a C3, C4, or Crassulacean Acid Metabolism (CAM) photosynthetic metabolism. Considering that each type of these plants typically grows at certain latitudes, the isotopic distribution of C may be, at first instance, indirectly exploited as a marker of origin. Since fractionation of C is expected to vary between the artificial feed used to rare aquaculture fish and the natural food of wild fish, its isotopic ratio may also be exploited to distinguish the production method of fish [72]. Indeed, isotopes of N are good indicators of the feeding regime of fish and of the position occupied by the fish in the food chain, thus being ideal markers of the production methods. Wild fish at higher trophic levels is in fact characterized by a greater enrichment in δ^15^N (^15^N/^14^N), and δ^15^N enrichment in artificial feeding given to farmed fish is expected to be significantly different compared to those present into the natural food eaten by wild fish [72].

In the present review, recent research in the field of multi-elemental analysis applied to edible tissues of fish and seafood was taken into consideration and reviewed. The scientific literature herein includes research articles pertinent to the topic of the present review and published between 2010 and 2020. Articles were retrieved from the Web of Science and Scopus databases (search terms: ‘fish,’ ‘seafood,’ ‘authentication,’ ‘elemental analysis,’ ‘elemental profile,’ ‘elemental fingerprinting,’ ‘chemometrics’).

**Figure 1:**
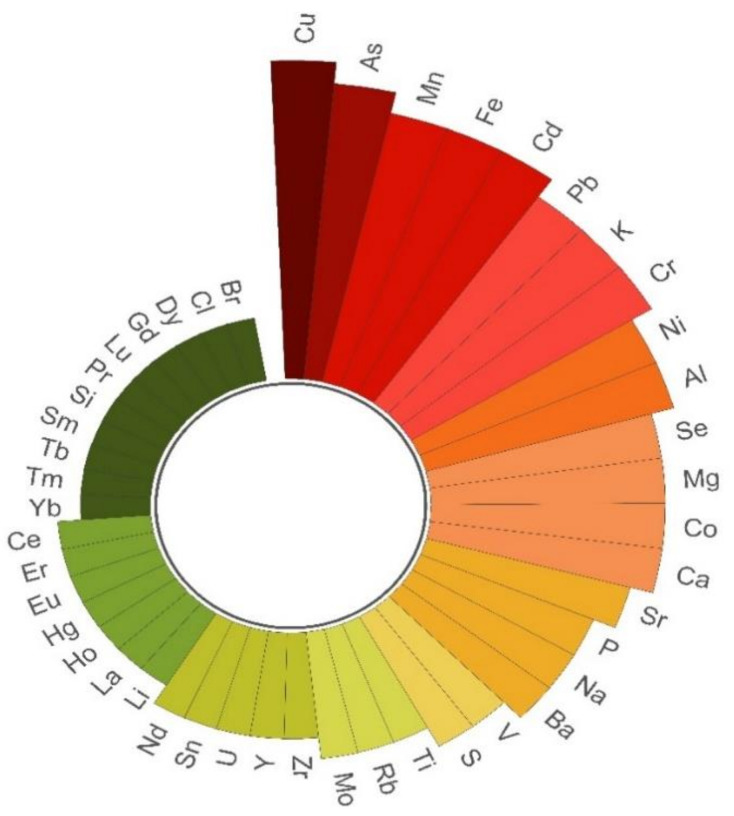
Radial bar chart showing the most widely used elemental markers in the works from the last 10 years dealing with authenticity and traceability of fish and seafood products. Data were elaborated from the scientific literature (published in 2010–2020) and collected from the Scopus and Web of Science search engines, using ‘fish,’ ‘seafood,’ ‘authentication,’ ‘elemental analysis,’ ‘elemental profile,’ ‘elemental fingerprinting,’ and/or ‘chemometrics’ as search terms.

For the sake of clarity, the next paragraphs are structured to enclose the same type of product. Therefore fish, mollusks (both bivalve and cephalopods), crustaceans, and echinoderms are discussed separately. The most frequently measured elemental markers of both geographical origin and method of production, retrieved from the reviewed scientific literature discussed below, are graphically shown in the radial bar chart reported in Figure 1. For a quick comparison, a summary overview of the methodological and technical aspects of the published works is given in Table 1. The concentrations of the elements measured in each work were deepened and provided in the Supplementary File (Table S2).

### 3.1. Fish

The maximum guarantee of transparency about the method of production, intended as catching wild fish or raising aquaculture fish, is of extreme importance, given that the two products have a differing economic value. In addition, certain farmed fish such as salmonids are reported to be more prone to accumulate environmental toxic substances, especially of organic nature [73], thus questioning the overall wholesomeness of these products. Tracing the geographical origin of aquaculture products may be, in some ways, more complicated than tracing that of wild-caught products. In fact, despite the fact that the feeding habits and prey availability for wild fish are highly variable and cannot be controlled, it should be emphasized that feeds used in aquaculture practices (which significantly affect mineral and trace element contents of fish tissues) are not only extremely variable in terms of composition but are frequently used worldwide to raise fish of different geographical origin [74], thus masking any discriminant potential of the elemental profile.

Despite these hurdles, different species of both wild and farmed salmons corresponding to king salmon (*Oncorhynchus tshawytscha*), coho salmon (*Oncorhynchus kysutch*), and Atlantic salmon (*Salmo salar*) were analyzed for their major and trace elemental content and isotope ratio profile of carbon (δ^13^C) and nitrogen (δ^15^N) in order to develop a model suited for their classification [61]. As for the type of employed tracers, it was verified that using elements or isotope ratios has no bearing on the overall performances of salmon classification, but on the contrary, the outcomes are strongly influenced by the number of samples employed to train the classification model as well as by the chosen classification algorithm. On that note, using machine learning algorithms as artificial neural networks (ANNs) and neural network bagging (NNB) gave a 94% and 92% correct classification rate, respectively, when applied to elements only, and 94% and 87% when using stable isotope ratios only [61].

The possibility of using rare earth elemental profile and/or light stable isotope ratios to identify fish production methods was also recently investigated for European sea bass *(Dicentrarchus labrax,* L.) samples [62]. In this case, the concentrations of lanthanum, europium, holmium, erbium, lutetium, and terbium elaborated by PCA and orthogonal partial least square discriminant analysis (OPLS-DA) did not impact the differentiation of wild from farmed specimens in contrast to light isotope ratios of carbon (δ^13^C) and nitrogen (δ^15^N), which had a higher influence. However, the authors verified that holmium and lanthanum, due to their natural variability in the marine environment, had a significant influence on the discrimination of the same samples by geographic origin. As a matter of fact, almost 89% of unlabeled samples from three different fishing areas in the Mediterranean Sea (used to test the validity of the developed model) were correctly discriminated [62].

The truthfulness of the label description of European seabass *(Dicentrarchus labrax,* L.) was also analyzed in another study which took into consideration the outputs obtained through the measurement of several parameters, corresponding to the biometric indices, fatty acids profile, analysis of 18 elements, and stable isotope ratios of carbon and nitrogen [48]. The method of production, the intensity of farming system, and the geographical provenance of sea bass were better discriminated using the fatty acids composition, while the use of elements alone outperformed compared to the other analytical data. Only the concentrations of Ca were in found to be significantly affected by feeding system and geographical origin, but the differences in fish tissues were not sufficient to achieve satisfying discrimination results, which settled around 79% for production method and 57% for the origin. On the contrary, stable isotope ratio data performed well in discriminating the production method of samples due to the strong influence of the feeding inputs on these parameters, but they were not able to classify samples according to provenance [48].

The merging of the results of multi-elemental and stable isotope ratios analyses has been successful also for the discrimination by origin and production method of Asian sea bass (*Lates calcarifer*) [49] and, when adding proximate composition, for the discrimination by origin of croaker [19]. Unlike the previously reported studies, XFR was the chosen technique to determine the elemental content of fish samples, mainly offering advantages in terms of the speed of operation. In this case, although the origin discriminant models for sea bass created by applying LDA or RF to stable isotope data were more accurate than those computed using elemental data only, isotopic analysis was less performant when used alone to predict both the origin and the production method of unknown samples, thus suggesting that information provided by elements is essential to achieve satisfying discrimination accuracy for the identification of geographical provenances [49].

One single attempt to discriminate the origin of freshwater cultured fish was found in the literature. In this case, fillets of channel catfish (*Ictalurus punctatus*) and hybrid catfish (*Ictalurus furcatus*) from 3 geographic areas were subjected to ICP-OES to measure a total of 11 elements [33]. Although the authors did not find a direct influence of water and feed used to raise the catfish on the final elemental composition of the fillets, the products were separated by origin, with 100% accuracy whether canonical discriminant analysis or *K*-nearest-neighbor analysis were used. Despite this, it should be noted that provenances considered in this study are of geopolitical rather than of geochemical nature. Therefore, the validity of the discrimination is limited by the fact that aquaculture catfish can be raised elsewhere in waters with an equivalent elemental composition [33].

### 3.2. Echinoderms and Crustaceans

Mislabeling of echinoderms has been poorly treated by the scientific community, probably because the consumption of these products, however high, is mainly limited to Asian countries. Two applications regarding the authentication of sea cucumber (*Apostichopus japonicus*) through elemental profiles have aimed at classifying the samples according to three [23] and five [63] sampling areas in China. However, these applications used a different number of elements, with 15 elements in the first case and to 39 in the second one. In both works, a stepwise-LDA was used to concomitantly sort elements by their relative importance in discrimination and build classification models. Concentrations of Al, Mn, Fe, Co, Ni, Cu, As, Se, Cd, and Hg were found to be appropriate to differentiate 100% of sea cucumbers in relation to the three sampling areas [23], while concentrations of Li, Na, Al, K, Co, Cu, Cd, and Sc made it possible to achieve 88% accuracy in differentiating samples originating from the five areas [63]. So, despite the higher number of elements measured in the second study, measuring a higher number of elements is not always a straightforward matter to achieve better discrimination results. If redundant or noise elements are not strictly evaluated and removed by proper statistics, models built using many elements as variables are likely to outperform, especially with an increasing number of origins to be identified.

Reviewing the literature, crustaceans emerged as the most frequently analyzed category of seafood products intended to be authenticated by their elemental composition (see Table 1). More specifically, six out of seven works analyzing the multi-elemental profile of crustaceans and taken into consideration in the present review dealt with the authentication of the origin of shrimps or prawns [22,25,64,65,66,68]. Among these, only two works concurrently investigated the possibility of using the same profile to address other problems, such as the production method and the species identifications [25,65].

The use of the elemental profile alone was demonstrated to be an optimal strategy to accurately assess the traceability of Pacific white shrimps (*Litopenaeus vannamei*) from different sampling sites in the USA [64] to differentiate shrimps obtained from Vietnam, Thailand, and India, which represent the biggest producing countries in the world [22]. When used in combination with light stable isotope ratios of carbon and nitrogen, the elemental profile was able to discriminate shrimps according to different sampling areas in China [64]. In general, despite the combination of major, minor, and trace elements (especially K, Mg, Na, P, Ca, Ba, Cr, Pb, Se, Si Cd, Co, and Zr), the elemental profile was successful in solving the origin discrimination problems in all cases. When concentrations of REEs were determined and used as discriminant variables, it was found that these elements had a greater analytical significance in determining the provenance of shrimps compared to other variables [64].

To some extent, the superiority of the element composition over stable isotope ratios of C and N to assess the traceability of shrimps was also demonstrated when farmed and wild samples of seven different biological species, obtained from nine sampling zones, were investigated [65]. Stable isotope analysis alone yielded to 100%, 71%, and 58% of samples to be correctly classified using LDA by production method, origin, and biological species, respectively. However, with an increasing number of samples into the models, the origin discrimination accuracy decreased or did not significantly increase. On the contrary, As, Cd, Pb, P and S concentrations alone showed greater accuracy in classifying samples by origin (94%) and species (74%) and, when merged with stable isotopes ratios, the two techniques showed the maximum discrimination power [65]. Similarly, both the production method and the origin traceability of prawn (*Penaeus monodon*) were assessed, with 100% accuracy when the multi-elemental profile and stable isotopes ratios were used complementarily [25].

Advantages of coupling elements and light stable isotope ratio analyses outputs to verify the exact provenances of high-value crustaceans are even more evident when powerful classification machine learning techniques are applied. The contents of Na, Mg, Al, K, Ca, Mn, Cu, Zn, Sr, and Ba, plus δ^13^C and δ^15^N measured on limited sample material and elaborated by means of SVM, allowed for the tracing of Chinese mitten crabs (*Eriocheir sinensis*) according to eight different geographical origins around China, with 100% and 97% accuracy in cross-validation and external validation, respectively [67].

### 3.3. Mollusks

To date, mollusks have appeared to be the least frequently studied aquatic products in terms of the evaluation of authenticity and fraud verification. This is particularly remarkable considering that, according to the latest available data, the worldwide supply of cephalopods and other mollusks has reached values of 3,535,732 tons and of 17,500,801 tons per year, respectively [75].

Historically, the elemental profiles of bivalve or cephalopods mollusks were employed to assess their geographical authenticity but performing such analyses on nonedible hard parts of the animals (e.g., shells, statoliths, beaks) [76,77] does not guarantee the possibility to apply the same methods to ready-to-cook products (eviscerated, beheaded, shelled), which are rising in popularity in international markets.

An interesting study used ICP-MS in combination with LDA, SIMCA, and ANNs to quantify and elaborate a total of forty elements in order to authenticate Galician mussels (*Mytilus galloprovincialis*) under the European Protected Designation of Origin (PDO) and protect the products from similar but lower-quality mussels [24]. A strong relation between element composition of PDO mussels and the geomorphology and lithology of the specific production zone, as well as with external contamination sources, was found. Whereas the Se, Zn, Pb, Co, Mo, Ag, and Ba elemental signature was attributed to the metabolic activity of the animals, the Ga, Zr, Eu, Lu, Th, and U signature was specifically related to mineralogical sources of the area, and the V, Cd, and Sb signature was related to the anthropogenic pollutant activities characterizing the area [24]. Keeping the complementary information provided by all these elements, PDO from non-PDO products were 100% accurately classified by LDA and SIMCA. On the contrary, the use of ANNs was found to be more effective in discriminating the five different sampling zones from which the PDO mussels were obtained.

In another work, particular attention was paid toward any effect that the seasonality had on the elemental composition of bivalves [69]. Since season variations were misleadingly reflected on the Mg, Rb, Pd, Cd, Sn, Ba, La, and Ce distribution into the mollusks, the authors were able to authenticate samples of Manila clams (*Ruditapes philippinarum*) using a different pattern of elements composed by Mg, Rb, Pd, Cd, Sn, Ba, La, and Ce, which, in contrast, was found to be more strongly linked to the geographical origin of clams [69].

As far as we know, no works oriented toward the evaluation of cephalopods mislabeling by measuring element composition of edible tissues such as mantles and fins are available. Nevertheless, the inorganic composition of ink derived from cuttlefish (commonly used in the Mediterranean and Japanese gastronomy) showed some potential ability to enclose geographical-related information [70]. Although no classification analysis was performed, some elements, such as Cr, Ni, V, Cd, Pb, As, and Hg, were significantly different among ink samples of cuttlefish (*Sepia officinalis*) of different sampling sites in the central Mediterranean Sea, suggesting that the contribution of the environmental pollution should be further investigated in these kind of studies to verify whether it can reveal actionable insights.

## 4. Why Are Aquatic Animals Ideal Candidates for Multi-Elemental Analysis?

The evaluation of the organic composition of foodstuffs continues to be the first choice when the identification of individual markers or patterns of markers for authenticity and traceability of fishery products is the main research goal. Nevertheless, measuring of a high number of organic components without carefully considering their origin, significance, sources of variations, and the general framework within which they are evaluated frequently puts their outright specificity as markers of origin into question. Indeed, the concentration and distribution of certain classes of organic compounds, such as fatty acids, peptides, and enzymes, are concurrently affected by so many aspects and circumstances that it is often challenging to univocally relate them to the sole species and geographic and/or farming origin. Pre-catch conditions, such as seasonality, climatic conditions, fishing period, fish size, fish physiology and metabolism, and fishing gear, as well as human’s post-catch manipulation and storage operations (storage temperature, packaging, lifetime of the product, and so forth), are just a few examples of factors affecting the organic composition of fish [9]. Similar considerations are valid also for inorganic constituents, but the correlation between the elemental compositions of fish tissues and the surrounding aquatic environment has been demonstrated to be more stable and consistent over time. Therefore, the probability that inorganic markers of fish origin are hidden by misleading factors may be considered lower compared to organic markers. Based on the concentrations found in the matrix, elements are normally categorized as major and minor (trace and ultra-trace) elements. A detailed definition has been reported only for trace elements, defined as those elements whose concentrations in the matrix are lower than 100 mg kg ^− 1^ [78], and which are mainly represented in food by B, Al, Fe, Mn, Ni, Cu, Zn, As, Se, Sr, and sometimes La and Ce. Consequently, major elements have mass fractions above 100 mg kg ^− 1^ (Na, Mg, P, K, Ca, Mg), while ultra-trace elements have mass fractions generally below 1 mg kg ^− 1^ [37] (Li, V, Cr, Co, Rb, Y, Zr, Mo, Ru, Pd, Cd, Sn, Ag, Cd, Sn, Sb, Cs, Ba, lanthanides, Hf, Re, Pt, Bi, Hg, Th, U, Hg). Rare earth elements (REEs), usually including Y, La, and lanthanides (Ce, Pr, Nd, Pm, Sm, Eu, Gd, Tb, Dy, Ho, Er, Tm, Yb, and Lu) [79], are emerging as very promising inorganic markers of fish authenticity, despite the fact that their quantification into foodstuffs is still limited to the very low abundance and consequent obstacles in quantification by modern instrumentations. Concentrations of REEs in both surface water and groundwater were found to vary significantly in relation to the geographical areas, with the Asiatic continent (and, in particular, China) showing the highest levels, followed by Europe, Africa, USA, and Australia [80]. These variations may be attributed both to the natural release of REEs from the parental soil (weathering of black shale is a common cause of increasing REEs composition in water) and to some anthropic activities (metallurgy, glass and ceramic industry, electronics) responsible for the REEs release into the aquatic environment and the consequent uptake by the aquatic fauna [81].

The overall major and minor elemental composition of fish is largely related to the elemental content of the eaten preys, vegetation, or fodder. In turn, the content of elements in animal and vegetable feeds is the result of the bioavailable elements which have been mobilized from the soil and which reflect the overall characteristics of the geographical area [12,82]. For example, as some alkaline metals (e.g., Rb and Cs) can be easily mobilized from the underlying soils, the probability that their incorporation into fish tissues is variable according to the geographical site is very high [83]. Other trace elements, such as B and As, naturally enter the aquatic environment from volcanic and geothermal activities [84,85]. Therefore, their concentrations in fish and seafood tissues may be exploited to discriminate animals from marine areas with specific geochemical characteristics. Moreover, concentrations of some major and trace elements, such as Li, Mg, Ca, Sr, Zn, Mn, and Cu, are strictly regulated by the salinity of the marine basin, and this characteristic makes them suitable to be potentially used for marine fish-tracing purposes [86]. In this setting, it is not unexpected that traceability studies concerning marine fish species are, to some extent, more standardizable, and thus more reliable compared to those dealing with freshwater species. Along with some concern deriving from the closeness to the anthropic environment, this aspect is attributable to the higher degree of dynamism of the marine systems compared to freshwater ones. Since this dynamism is biologically, chemically, and physically controlled, a more uniform element concentration from both a temporal and spatial point of view can therefore be found in the marine environment, especially in open ocean waters. Nevertheless, when performing authentication studies, consideration must be given to the fact that the distribution ratio of certain elements between fish tissues and seawater is altered by the metabolic activity of animals [74]. Specifically, the uptake of many essential elements, such as Na, K, Mg, and Ca, is metabolically regulated by the same fish, since it is necessary for regulating physiological functions. Hence, the potential for variation of these elements in relation to origin is masked by physiological ‘noise’ [87]. Consequently, they are hardly ever used in fish authentication studies.

Finally, a greater compositional heterogeneity is encountered in waters of coastal areas compared to deep seawaters, where the proximity of anthropic releasing sources leads some trace and ultra-trace elements to be variably introduced into the marine environment. Nickel, zinc, arsenic, lead, mercury, and cadmium are well known for their higher concentrations along shorelines [88], since they are derived from certain agricultural practices or industrial activities. On the other side, fish and seafood are not able to physiologically regulate the concentration of these nonessential (and often toxic) elements, which, consequently, are passively accumulated into the animal’s tissues. If properly evaluated, anthropic elements can also be used for origin authentication purposes [89].

To conclude, although introduced through different sources, elements can be successfully employed as authenticity markers of fish and seafood if the same introduction sources are systematic, identifiable, manageable, and suggestive of the geographical origin or production process [74]. In this context, when dealing with authentication of transformed fish products, particular attention should be paid against the introduction of elements from the production chain. These obstacles may be often overcome by comparing the effective concentrations of elements in the final products with those found along as many stages as possible of the transformation process, so as to be able to verify whether distribution trends are retained along the production stages [74].

## 5. Final Remarks and Conclusions

The application of element profiling approaches to fish and seafood products has been gaining momentum, and the scientific community has been working on the optimization of both existing instrumentations for multi-elemental analysis and algorithms for statistical analysis. The greater thrust has come from advances in chemometrics and machine learning techniques, which now provide great support to the identification of maximum relevant chemical information from large datasets not otherwise accessible.

From the analysis of the literature presented in this review, it is clear that the discrimination of the geographical origin has been the most frequently discussed authenticity topic, while other aspects, such as the farming systems, have been overlooked. In addition, crustaceans have emerged as the most frequently investigated category of products, while less emphasis has been placed on fish, echinoderms, and mollusks, especially cephalopods, probably due to difficulties in drawing up an adequate sampling plan to build representative datasets. Regarding the statistical data treatment, PCA and LDA have been more widely used, while machine learning algorithms have been neglected, despite their great potential in discovering hidden discriminant patterns among data.

As for the selected methodologies, ICP-MS, followed by ICP-OES, has been the first choice, accounting for the vast majority of the published research. Especially in the last years, ICP-MS has been gaining popularity within the scientific community because it is less complicated, less expensive, and undoubtedly the fastest and most universal trace element technique commercially available today. This is mainly due to the advances in collision/reaction cell technology, which offers an effective way to reduce spectral effects from different polyatomic ions. Quadrupole mass spectrometers, in particular, are increasingly being used and, until recently, it seemed impossible that a single technique would fit perfectly to the needs of all the laboratories. For this reason, these instruments are expected to supersede most of the ICP-OES and AAS applications in the near future. In addition, it may be expected that various solid-sampling techniques, such as ETV-ICP-MS, LA-ICP-MS, and XRF, may succeed more in the field of food authentication, with the advantage of a reduced sample preparation.

Another peculiarity emerging from the published literature is the tendency to couple element profiles of fish and seafood with other analytical parameters, especially stable isotopes of carbon, hydrogen, and nitrogen, which has probably been motivated by the need to increase the accuracy of discrimination. Despite the benefits deriving from the fusion of complementary or synergistic information, it is worth highlighting that multi-elemental analysis may be sufficient to achieve equivalent results with an optimal cost-performance ratio.

Looking forward, the increased use of ICP-MS-hyphenated techniques for elemental speciation and ICP-MS/MS for interference-free determination and isotope ratio measurement would represent a turning point for the high-throughput analytical characterization of complex matrices such as food. Nevertheless, the reduction of the cost of the equipment for multi-elemental analysis would certainly be desirable to further encourage the spreading of multi-elemental analytical approaches in a different context from that of the specialized laboratories dealing with food surveillance. Before getting to this point, the validity and robustness of elemental markers to ascertain fish and seafood authenticity must be increased. Further work on these issues is therefore encouraged in order to integrate information relating to any possible variable influencing the inorganic profile of fishery products with the elemental information relating to the origin into adequately defined reference databases. At the same time, continuous technological improvements, as well as the shift toward a progressive miniaturization of the instruments, may be a major turning point, helping to concomitantly monitor health risks associated with the occurrence of toxic metals such as cadmium, lead, mercury, and arsenic, and to meet the demand for cost-effective and energy- and reagent-saving instruments.

## Figures and Tables

**Table 1:** Overview of the literature dealing with multi-elemental profile for fish and seafood authenticity verification.

Product	Classification Objective	Input Data	Technique for Elemental Analysis	Elements	Data Analysis	Validation	Reference
*Fish*							
Salmon	Production method	Elemental profile Stable isotope ratio	ICP-OES	As, Ba, Be, Ca, Cd, Co, Cr, Cu, Fe, K, Mg, Mn, Na, Ni, P, Pb, Sr, Ti, Zn	PCA, CDA, LDA, QDA, ANNs, PNNs, NNB	Cross-validation External validation	[61]
Catfish	Geographical origin	Elemental profile	ICP-OES	Al, Ca, Cr, Cu, Fe, K, Mg, Na, P, S, Zn	PCA, CDA, k-NN	Cross-validation	[33]
Croacker	Geographical origin Seasonality	Elemental profile Stable isotope ratio Proximate composition	EDXRF	As, Br, Ca, Cd, Cl, Cu, Fe, Hg, K, Pb, Rb S, Se, Zn,	PCA	–	[19]
European seabass	Geographical origin Production method	Elemental profile Stable isotope ratio Biometric measures Fatty acids	ICP-OES	As, Ca, Cd, Co, Cr, Cu, Fe, Hg, K, Mg, Mn, Na, Ni, P, Pb, S, Se, Zn	PCA	Cross-validation	[48]
Asian seabass	Geographical origin Production method	Elemental profile Stable isotope ratio	XRF	Al, As, At, Bi, Br, Ca, Cd, Cl, Cr, Cu, Fe, Hf, K, Mg, Mn, Nd, Ni, P, Pb, Rb, S, Sb, Se, Si, Sn, Sr, Ti, U, Y, Zn, Zr	PCA, LDA, RF	Cross-validation External validation	[49]
European seabass	Geographical origin Production method	Element profile Stable isotope ratio	ICP-MS	Er, Eu, Ho, La, Lu, Tb	PCA, OLPS-DA	Cross-validation External validation	[62]
*Echinoderms*							
Sea cucumber	Geographical origin	Elemental profile	ICP-MS	Al, As, Cd, Co, Cr, Cu, Fe, Hg, Mn, Mo, Ni, Pb, Se, V, Zn	PCA, CA, LDA	Cross-validation	[23]
Sea cucumber	Geographical origin	Elemental profile	ICP-OES ICP-MS	Ag, Al, As, Ba, Bi, Ca, Cd, Ce, Co, Cr, Cu, Dy, Er, Eu, Fe, Gd, Ho, K, La, Li, Lu, Mg, Mn, Na, Nd, Ni, Pb, Pr, Sc, Se, Sm, Sn, Sr, Tb, Tm, V, Y, Yb, Zn	PCA, LDA	Cross-validation	[63]
*Crustaceans*							
Pacific white shrimp	Geographical origin	Elemental profile	ICP-OES	Al, As, Ba, Ca, Co, Cr, Cu, Fe, K, Mg, Mn, Mo, Na, Ni, P, S, Se, Ti, Zn, Zr	PCA, CDA, k-NN	Cross-validation	[64]
Shrimps	Geographical origin Production method Species	Elemental profile Stable isotope ratio	ICP-OES ICP-MS	As, Cd, P, Pb, S	PCA, CA, LDA, k-NN	Cross-validation	[65]
Prawns	Geographical origin	Elemental profile Stable isotope ratio	ICP-MS	Al, As, B, Cd, Co, Cr, Cu, Fe, Hg, K, Li, Mn, Mo, Ni, Se, Sr, Ti, V, Zn	PCA, CDA	Cross-validation	[66]
Pacific white shrimps	Geographical origin	Elemental profile	ICP-OES	Al, As, B, Ba, Ca, Cd, Co, Cr, Cu, Fe, K, Mg, Mn, Na, Ni, P, Pb, S, Se, Si, Ti, Zn, Zr	PCA, CDA, S-LDA	Cross-validation	[22]
Chinese mitten crab	Geographical origin	Elemental profile Stable isotope ratio	ICP-MS	Al, Ba, Ca, Cu, K, Mg, Mn, Na, Sr, Zn	LDA, SVM	Cross-validation External validation	[67]
Pacific white shrimps	Seawater vs. Freshwater	Elemental profile Stable isotope ratio	ICP-MS	Ag, Al, As, Ba, Cd, Ce, Co, Cr, Cs, Cu, Dy, Er, Eu, Fe, Ga, Gd, Ho, Li, Lu, Mn, Nd, Ni, Pb, Pr, Rb, Sm, Sr, Tb, Th, Tm, U, V, Y, Yb, Zn	PCA, CDA, S-LDA	Cross-validation	[68]
Black tiger prawn	Geographical origin Production method	Elemental profile Stable isotope ratio	XRF	Al, As, At, Bi, Br, Ca, Cd, Cl, Cr, Cu, Fe, Hf, K, Mg, Mn, Nd, Ni, P, Pb, Rb, S, Sb, Se, Si, Sn, Sr, Ti, U, Y, Zn, Zr	LDA, RF	Cross-validation External validation	[25]
*Mollusks*							
Mussels	Geographical origin	Elemental profile	ICP-MS	Ag, As, Ba, Cd, Ce, Co, Cr, Cu, Dy, Er, Eu, Ga, Gd, Ho, La, Lu, Mn, Mo, Nb, Nd, Ni, Pb, Pr, Rb, Sb, Se, Sm, Sn, Sr, Ta, Te, Th, Tl, Tm, U, V, Y, Yb, Zn, Zr	LDA, SIMCA, ANNs	Cross-validation	[24]
Manila clams	Geographical origin	Elemental profile	ICP-MS	Al, As, Ba, Cd, Ce, Co, Cs, Cu, Fe, K, La, Mg, Mn, Na, Mo, Pb, Pd, Rb, Sb, Se, Sr, Sn, U, V, Zn	S-LDA	Cross-validation	[69]
Cuttlefish (ink)	Geographical origin	Elemental profile	ICP-MS	As, Ca, Cd, Co, Cr, Cu, Fe, Hg, K, Mg, Mn, Mo, Na, Ni, P, Pb, V, Zn	PCA	–	[70]

ANNs = artificial neural networks. CDA = canonical discriminant analysis. EDXRF = energy dispersive X-ray fluorescence spectroscopy. ICP-OES = inductively coupled plasma optical emission spectroscopy. ICP-MS = inductively coupled plasma mass spectrometry. k-NN = k-nearest neighbors. LDA = linear discriminant analysis. NNS = neural network bagging. PCA = principal component analysis. PNNs = probabilistic neural networks. QDA = quadratic discriminant analysis. RF = random forest. SIMCA = soft independent modelling of class analogy. S-LDA = stepwise-linear discriminant analysis. XRF = X-ray fluorescence spectroscopy.

## Supplementary Materials

The following are available online at https://www.mdpi.com/2304-8158/10/2/270/s1, Table S1: A summary of the advantages and disadvantages of the main each analytical methodology examined in the present review, Table S2: Concentrations of the elements (means and standard deviations in brackets, concentrations expressed as mg kg^−1^) in the reviewed studies.
