# Supplementary material for: Advances in Troubleshooting Fish and Seafood Authentication by Inorganic Elemental Composition
Source: Foods. 2021 Jan 29;10(2):270. doi: 10.3390/foods10020270 (PMC7912245; doi:10.3390/foods10020270)
Supplement: Supplementary file 1 [file foods-10-00270-s001.pdf]

SUPPLEMENTARY FILE

Advances in Troubleshooting Fish and Seafood Authentication by Inorganic Elemental Composition

Maria Olga Varrà, Sergio Ghidini, Lenka Husáková, Adriana Ianieri, and Emanuela Zanardi

Table S1. A summary of the advantages and disadvantages of the main each analytical methodology examined in the present review (references to specific studies using these techniques are provided in the Section 2 of the main text).

Technique	Advantages	Drawbacks
FAAS (flame atomic absorption spectroscopy)	• quantification of nearly 70 elements;<br>• high selectivity and specificity;<br>• detection limits typically in the range of 100s μg kg^{-1};<br>• simple instrumental arrangement;<br>• low cost of the equipment .	• single-element determinations;<br>• working dynamic range 3-4;<br>• usually destructive and time-consuming sample preparation;<br>• often using concentrated acids;<br>• ionization buffers (Cs, Li or K) usually required to minimize the ionization interferences when analyzing easily ionized elements;<br>• detection of non metals (S, N and halogens) not achievable;<br>• poor detection limits for refractory elements;<br>• the use of a flame with reducing or oxidizing character influencing the atomization processes.
FOES (flame optical emission spectroscopy)	• simple, inexpensive and sensitive;<br>• better detection limits for e.g. alkaline and alkaline earths than FAAS;<br>• excellent sensitivity also for some transition metals (including Fe, Mn, Cu or Zn);<br>• typical detection limits below 100s ppb;<br>• relatively free of interferences from other elements;<br>• ability to operate at lower temperatures.	• usually arranged for a single analyte measurement or multi-element analysis in a rapid sequence;<br>• low temperature flame not suitable for elements others than alkali and alkaline earths metals;<br>• narrow concentration ranges (the samples must be greatly diluted);<br>• liquid samples usually needed;<br>• susceptible to chemical and physical effects and ionization interferences;<br>• possible issues related to sensitivity and cost.

(Continued)

Table S1 *(Continued).*

Technique	Advantages	Drawbacks
ET-AAS[a)] (electrothermal atomic absorption spectroscopy)	• versatile and highly sensitive;<br>• detection capability in the sub-ppb range;<br>• microliter-sized samples suffiecient;<br>• detection limits in the range of 0.01–0.1 μg L^{-1} (ppb);<br>• samples can be introduced into graphite furnace as solution;<br>• possibility of using slurry or direct solid sampling;<br>• excellent robustness of atomisers for the elimination of the matrix constituents;<br>• low purchase price and running costs;<br>• calibration can be performed with aqueous standards (even for slurry samples);<br>• possibility of using chemical modifiers to improve the analytical figures of merit and interference suppresion.	• one measurable element at a time;<br>• working dynamic range 2-3;<br>• homogenization of the sample is crucial for microanalysis of solid samples;<br>• efficient matrix elimination procedure required in the atomizers so that the matrix does not influence the analytical signal;<br>• knowledge of the atomisation mechanisms and the physico-chemical properties of the analyte is essential.
HR-CS-ETAAS (high-resolution continuum source atomic absorption spectroscopy)	• multielemental and simultaneous determination;<br>• high analytical performance;<br>• excellent light throughput and analytical sensitivity;<br>• extended measurement range of up to 5 orders of magnitude;<br>• no need for lamp changes;<br>• determination of metals and unique determination of non-metals (P, S, halogens, etc.) via diatomic molecular monitoring;<br>• isotopic analysis;<br>• possibility to analyse solid, complex and difficult-to-decompose samples;<br>• interference-free analysis (high-resolution optics and advanced correction algorithms dealing with complex spectral interferences, such as structured backgrounds, visualization of high-definition absorption spectra in both 2D and 3D for interference evaluation).	• non-spectral interferences relatively common;<br>• chemical modifiers usually required for interferences overcoming;<br>• higher purchace price compared to ICP-OES instrumentation.
HG-AAS (hydride generation atomic absorption spectroscopy)	• ppt detection limits for elements such as As, Bi, Sn, Sb, Te, Se, Ge and Hg;<br>• better precision and enhanced selectivity due to the reduction of interfering species;<br>• few and well known interferences;	• single-element analysis;<br>• the relatively few analytes forming hydrides to which the technique can be applied;<br>• potential interferences resulting from the presence of hydride-forming elements in the matrix which can influence the results;

(Continued)

Table S1 *(Continued).*

Technique	Advantages	Drawbacks
HG-AAS (hydride generation atomic absorption spectroscopy)	• preconcentration easily implemented;<br>• capabilities for rapid automation;<br>• possibility to perform speciation analysis;<br>• simplicity, high cost-effectiveness and robustness.	• auxiliary reducing reagents and/or masking agents usually required;<br>• complete sample decomposition or extraction usually required;
CV-AAS (cold vapour atomic absorption spectroscopy)	• detection limits in the single-digit ppt range;<br>• dynamic range of 2-3 orders of magnitude;<br>• the mercury analyte is removed from the sample matrix; reducing the potential for matrix interference;<br>• speciation analysis.	• relatively slow and laborious;<br>• appropriate sample pre-treatment often required (e.g., complete acid digestion for total-Hg measurement or selective extraction for methyl-Hg quantification);<br>• oxidation step (converting all analyte present to Hg (II)) followed by a chemical reduction reaction required before the measurement;<br>• additional preconcentration of analyte by amalgamation on gold or silver traps for Hg contents lower than mg kg^{-1};<br>• large reagent consumption;<br>• detection limits limited by reagents contamination.
DMA[b)] (direct mercury analysis)	• fast, cost-effective, sensitive, easy and safe;<br>• direct determination of total mercury without the need for acid digestion or other wet chemistry sample treatment or concentration steps;<br>• analytical times lower than 5 minutes;<br>• matrix-independent results, both solid and aqueous samples can be analysed with equal efficiency;<br>• no hazardous chemicals required;<br>• detection limit lower than 0.01 ng Hg;<br>• working range up to 500 ng Hg;<br>• reliable results over a wide content range;<br>• high robustness;<br>• one year calibration cycle sufficient;<br>• small quantity of sample (<10 mg) required.	• single-purpose analysis;<br>• non-homogeneous samples must be carefuly homogenized (or digestion of a larger quantity of sample required).
AFS (atomic fluorescence spectrometry)	• highly sensitive trace detection;<br>• dynamic working range of xx orders of concentration;<br>• linear dynamic range of 4-5 orders of magnitude;<br>• good sensitivity;	• extensive preparation;<br>• spectral interferences generated by atomiser emissions and source scatter;

(Continued)

Table S1 *(Continued).*

Technique	Advantages	Drawbacks
AFS (atomic fluorescence spectrometry)	• detection limits below μg L^{-1};<br>• sensitivity for several elements to the parts per trillion (ppt) level;<br>• wide linear calibration range, from ugL^{-1} to mg L^{-1};<br>• ideal detection technique for speciation studies concerning hydride forming elements (mainly As, Se and Sb) and Hg;<br>• lower acquisition and running costs compared to to ICP techniques, robustness and ease of opeartion.	• poor senistivity for certain elements (absorbing or emitting in the visible region);<br>• quenching when excited atoms collide with other molecules in the atomisation sources;<br>• source scatter and atomizer emission causing spectral interferences;<br>• chemical interferences result from various chemical processes during atomisation that reduce the population of free atoms.
ICP-OES[c)] (inductively coupled optical emission spectrometry)	• robust and rapid;<br>• multi-element analysis of solutions or slurries;<br>• satisfactory accuracy and precision for analysis up to 70 elements (especially with the use of internal standards);<br>• detection limits down to ppb;<br>• linear dynamic range of 7-8 orders of concentration;<br>• detection limits of tens of ppt (pg/mL) or below;<br>• ultrasonic nebulizer offers enhanced detection limits up to 10x (or greater);<br>• less expertise to operate;<br>• versatility regarding different types of samples;<br>• sample introduction systems with fast washout times and simultaneous elemental detection;<br>• sample measurement times of less than 1 min;<br>• possibility for semiquantitative analysis (identification of elements whose concentrations are enough to cause matrix effects or spectral overlaps;<br>• quantification of non metals (such as S, N, C), and halogens (e.g. I, Cl, Br);<br>• better detection limits for refractory elements than e.g. AAS;<br>• almost free from chemical interferences.	• usually time consumming dissolution step with different analytical reagents;<br>• higher detection limits than ICP-MS;<br>• spectral interferences (from the background emission shifts and/or the overlap of lines emitted from other elements) more serious and difficult to eliminate than in AAS;<br>• appropriate inter-element correction equations and multi-component spectral fitting to be adopted;<br>• elements at concentrations higher than 500 ppm can affect sensitivity;<br>• less useful for the analysis of some elements such as As and Se (high ionization potentials resulting in poor LODs).
MP-OES (microwave plasma optical emission spectrometry)	• simultaneous multi-analyte determination of major and minor elements;<br>• working range from ppm to weight percent (wt. %) for most elements;<br>• Ar gas not required (nitrogen extracted directly from air);	• detection limits of elements with high excitation energies (such as As, Se, Cd, P, Sb, Se, and Zn) are poorer than those provided by ICP-OES;<br>• detection limits for many elements not yet competitive with ICP-OES;

(Continued)

MP-OES (microwave plasma optical emission spectrometry)	• dynamic working range of 4-5 orders of concentration;<br>• detection limits (down to sub-ppb levels);<br>• operating and purchasing costs lower than those of high-end techniques like ICP-QMS and ICP-QQQ;<br>• high-performance, robustness and reliability;<br>• better detection limits, speed of analysis and larger linear dynamic range compared to conventional flame AAS.	• limited number of manufacturers supplying instruments commercial MP-OES instruments.
Q-ICP-MS (quadrupole-inductively coupled plasma mass spectrometry)	• highly sensitive, powerful and a fast;<br>• multielement capability;<br>• the broadest element coverage (mass range 2–260 amu);<br>• the widest dynamic range (up to 11 orders-of-magnitude) from sub-ppt to percent-level;<br>• the best detection limits (lower than ppt);<br>• trace elements and majors measurable in the same run;<br>• control of spectral interferences via the collision/reaction cells for the effective removal of polyatomic interferences in reaction mode;<br>• high robustness allowing matrix levels up to several percent total dissolved solids to be analyzed routinely;<br>• ability to discriminate between isotopes;<br>• suitability as a selective detector in hyphenated methods for elemental speciation.	• spectral interferences (isobaric overlap, overlap from polyatomics, and doubly charged) must be accounted for complex sample matrices;<br>• nonspectral interferences can occur (e.g, transport effects, ionization interferences, ion sampling effects) to be controlled with internal standards and standard additions;<br>• suitable internal standards to be chosen with care;<br>• sensitivity for certain analyte ions can be significantly reduced using reactive/collision cell gases;<br>• purchase and maintenance costs higher than ICP-OES.
ICP-QQQ (triple quadrupole inductively coupled plasma mass spectrometry)	• among the most powerful and flexible multi-element analyzer;<br>• advanced interference removal and high accuracy even in challenging analytical applications;<br>• full and semi-quantitative multi-element screening with the highest matrix tolerance;<br>• outstanding detection limits and a dynamic working range of 11 orders of concentration;<br>• little sample preparation and processing;<br>• fast, accurate, and consistently reproducible results (even for trace metals);<br>• resolution of isobaric overlaps, beyond capability of high-resolution ICP-MS;<br>• lowest detection limits even for difficult elements (S, Si, P, etc.).	• high instrument cost;<br>• complex setup and operations;<br>• need for a skilled operator to perform method development;<br>• high pressure acid digestion usually required before analysis.

(Continued)

Table S1 *(Continued).*

Technique	Advantages	Drawbacks
HR-ICP-MS[d)] (high resolution inductively coupled plasma mass spectrometry)	• class-leading elimination of spectral interferences by resolving spectral overlaps;<br>• ultra-sensitive multi-element analyses;<br>• full- or semi-quantitative multi-element screening;<br>• quantification at single digit ppq (pg L^{-1}) levels;<br>• potentially generate elemental fingerprints of a much higher complexity than Q-ICP-MS;<br>• good precision reported for isotope ratio;<br>• higher resolution or higher sensitivity.	• acid digestion, closed-vessel microwave digestion or high pressure ashing needed;<br>• internal standards to be incorporated for each sample at known concentrations for the desired element(s) to compensate for any variation in the intensity of the element signal;<br>• high costs of the equipment and operations.
TOF-ICP-MS (time-of-flight inductively coupled plasma mass spectrometry)	• simultaneous analysis of the entire elemental mass spectrum<br>• high sample throughput;<br>• multi-element fast measurement of transient signals;<br>• simultaneous internal standardization as well as isotope ratios measurements for all elements with precision better than 0.1 % RSD;<br>• enhanced resolution compared with Q-ICP-MS for suppression of matrix ions;<br>• advanced octopole collision cell technology for superior interference management provided;<br>• small volume of samples required;<br>• sensitivity for single element analysis is similar to Q-ICP-MS.	• resolution is not adequate to resolve the most practical interferences occurring during the real samples analysis;<br>• Q-cell technology for suppression of matrix ions is available only for current generations of instruments (for older devices difficult isobaric interferences must be corrected by extensive and/or complex sample preparation techniques or by mathematical corrections);<br>• sensitivity as much as one order of magnitude poorer than Q-ICP-MS;<br>• lower sensitivity than HR-ICP-MS.
IRMS (isotope ratio mass spectrometry)	• precise and sensitive measuring of isotopic signatures;<br>• highly suitable for stable-isotope analysis of H, C, N, O and S (superior to Q-ICPMS and HR-ICP-MS);<br>• sub ug g^{-1} concentration range;<br>• high level of precision (lower than 0.02%);<br>• excellent linearity and stability;<br>• small amounts of samples required;<br>• possible multiple-collector analysis;<br>• high-quality peak shapes;<br>• important role for bulk-tissue analysis and developments of authentication procedures.	• analysis of only a very limited number of elements;<br>• samples must be converted into gas (e.g., H_2, N_2, CO_2, CO, or SO_2) before analysis and introduced via gas chromatography into a mass spectrometer.

(Continued)

Table S1 *(Continued).*

Technique	Advantages	Drawbacks
MC-ICP-MS (multi-collector inductively coupled plasma mass spectrometry)	• precise and accurate isotope ratio analysis;<br>• stable-isotope analysis of elements unsuitable for traditional IRMS (e.g., B, Mg, Sr and Pb);<br>• in-situ isotopic measurements in solid materials when coupled with laser ablation;<br>• analysis of most of the elements of the periodic table (including those with high ionization potential);<br>• greater speed of analysis, higher throughput of samples, relatively simple sample preparation and better accessibility to laboratories compared to thermal ionisation mass spectrometry (TIMS).	• purchase price extremely high;<br>• operational costs of analyses significantly higher than IRMS;<br>• samples need to be chemically purified to achieve the highest precision and accuracy levels;<br>• plasma instability can limit precision;<br>• transmission of ions lower than with TIMS;<br>• much larger isotopic fractionation/mass bias and poorer precision and reproducibility than TIMS.
TIMS (thermal ionisation mass spectrometry)	• very precise determination of isotope ratios lower than 0.01%;<br>• alternative to MC-ICP-MS for stable isotope analysis of high mass elements;<br>• data commonly obtained on extremely small samples (e.g., down to the nanogram level);<br>• lower and more consistent average mass fractionation;<br>• use of single element solutions to eliminate isobaric interferences;<br>• easily automated operation;<br>• near 100% transmission of ions from source to collector;<br>• unlike ICP-MS, the TIMS source does not produce isobaric interferences that can cause inaccuracies.	• sample throughput very low;<br>• labor intensive and time consuming sample preparation steps needed to ensure high quality chemical separation of the analyte (to correct for isotopic fractionation);<br>• mass fractionation correction is limited to elements with 3 or more isotopes, of which at least 2 are particularly stable in the ion source;<br>• possible change in isotopic composition during the measurement;<br>• incomplete isotope (elemental) coverage;<br>• species with high ionization energy can be analysed more effectively with MC-ICP-MS.
ETV-ICP-OES (electrothermal vaporisation inductively coupled optical emission spectrometry	• rapid and direct analysis of small amount of liquid, slurry, and solid samples;<br>• multielement analysis;<br>• possibility to quantify major, minor and trace elements;<br>• high dynamic range;<br>• no hazardous chemicals to purchase;<br>• sensitivity at the ppb levels;<br>• sample intake from few to few tens of milligrams;<br>• tolerable amounts of matrix elements higher than for ETV-ICP-MS.	• risk of high variance of the results due to a small sample intake and material inhomogeneity;<br>• less sensitive than ICP-MS in most cases;<br>• lack of certified reference materials;<br>• the use in routine laboratories is still limited.

(Continued)

Table S1 *(Continued).*

Technique	Advantages	Drawbacks
ETV-ICP-OES (electrothermal vaporisation inductively coupled optical emission spectrometry	• rapid and direct analysis of small amount of liquid, slurry, and solid samples;<br>• multielement analysis;<br>• possibility to quantify major, minor and trace elements;<br>• high dynamic range;<br>• no hazardous chemicals to purchase;<br>• sensitivity at the ppb levels;<br>• sample intake from few to few tens of milligrams;<br>• tolerable amounts of matrix elements higher than for ETV-ICP-MS.	• risk of high variance of the results due to a small sample intake and material inhomogeneity;<br>• less sensitive than ICP-MS in most cases;<br>• lack of certified reference materials;<br>• the use in routine laboratories is still limited.
ETV-ICP-MS (electrothermal vaporisation inductively coupled plasma mass spectrometry)	• direct determination of contaminants in solids at trace and ultra-trace levels;<br>• detection limits of μg kg^{-1};<br>• no dissolution steps;<br>• risk of contamination and analyte loss considerably reduced;<br>• increased sensitivity;<br>• shorter times of analysis;<br>• small amounts of sample required;<br>• minimization of the use of hazardous reagents;<br>• the use of chemical modifiers can drastically reduce interferences from the matrix;<br>• accurate quantification less problematic compared to LA-ICP-MS;<br>• by means of thermal pre-treatment the bulk of the matrix is removed (the matrix effects are prevented or at least reduced to a large extent);<br>• accurate quantification can be usually accomplished by means of aqueous standard solutions.	• variability of transport efficiency when the elements present different volatilities;<br>• the limited amount of sample that can be analyzed may reduce reproducibility;<br>• memory and matrix effects;<br>• efficient vaporization and subsequent transport of the analyte to the ICP-MS is required;<br>• more prone to matrix effects compared with ET-AAS;<br>• transport of the analyte from the furnace to the ICP is often affected by the presence of the sample matrix, making sometimes quantification on the basis of aqueous standards difficult;<br>• the transient nature of the ETV signal and the scanning nature of the Q-ICP-MS typically only allow for the determination of no more than five elements simultaneously;<br>• very limited possibilities for spatially resolved analysis compared to LA-ICP-MS;<br>• not widely used in routine practice.
LA-ICP-MS (laser ablation inductively coupled plasma mass spectrometry)	• direct analysis of solids with no or minor sample pretreatment;<br>• multielement analysis in a concentration range covering up to 9 orders of magnitude;<br>• excellent sensitivity with LODs on the order of μg kg^{-1};<br>• only picograms and femtograms of the sample are consumed during the analysis;	• highly matrix-dependent;<br>• accurate quantification is not an easy task;<br>• lack of reliable procedures for external calibration;<br>• standards with a composition that is closely matched to the samples have to be used;

(Continued)

Table S1 *(Continued).*

Technique	Advantages	Drawbacks
LA-ICP-MS (laser ablation inductively coupled plasma mass spectrometry)	• impact of polyatomic interference caused by solvents reduced;<br>• considerably faster with less workload than techniques involving conventional digestion;<br>• mitigation of the contamination risk due to the dissolution procedure;<br>• no chemicals required and reduction of the amount of waste generated;<br>• suitable for mapping trace elements in different tissue types as well as for elements depth profile analysis.	• when CRM is not available, a suitable standard materials have to be prepared in-house;<br>• compact samples for analysis are required (powdered materials must be fixed onto a substrate or converted to a compact sample by e.g. pelletization, which reduces sensitivity and increases preparation times).
LIBS (laser induced breakdown spectroscopy)	• multielement analysis of solid samples with a broad elemental coverage;<br>• concentration ranges extending from major to trace elements;<br>• minimal sample preparation;<br>• extremely fast measurement time (usually a few seconds) for a single spot analysis, in contrast to laser ablation;<br>• coupling with another technique is not mandatory;<br>• no consumption of gases;<br>• possible in situ analysis;<br>• fast acquisition;<br>• operation at ambient atmosphere;<br>• no restrictions in the detection of light elements when compared with XRF;<br>• microscopic-scale resolution;<br>• capability of LIBS imaging to characterize complex samples;<br>• small amounts of material consumed;<br>• possibility of using portable instruments;<br>• far safer than XRF.	• quite high detection limits at the ppm scale for most elements;<br>• low reproducibility and repeatability;<br>• matrix effects, insufficient reproducibility, and nonlinear calibration;<br>• limitations in the availability of reference materials with a multitude of certified trace elements.
XRF (X-ray fluorescence spectroscopy)	• can be used to detect most of the elements in the periodic table ranging from Na to U (and even higher Z elements);<br>• well-suited for bulk chemical analyses of major elements and of trace elements (higher than 1 ppm);<br>• high analytical throughput, non-destructiveness, in situ analysis;	• high LODs;<br>• quantitative analysis not easy due to matrix effects;<br>• relatively large samples required;<br>• need for homogeneous and representative samples;<br>• the geometry of the sample can affect analysis;

(Continued)

Table S1 *(Continued).*

Technique	Advantages	Drawbacks
XRF (X-ray fluorescence spectroscopy)	• times for chemical compositions determinations rarely longer than one minute;<br>• little or no residue generated;<br>• possibility of in situ analyses;<br>• no risk of loss of elements during sample-handling operations;<br>• absence of contamination from reagents;<br>• wide dynamic concentration range (from ppm to tens percent);<br>• quantitative analysis using fundamental parameters without standards or calibration curves;<br>• cost-effectiveness for processing large numbers of samples;<br>• the product can be analysed from its packaging;<br>• drying and pelletising of the samples may not be necessary;<br>• acceptable quantitative results can be derived by simple homogenisation of the sample prior to analysis;<br>• highly accurate determinations for major elements.	• well-characterized and compositionally similar standard materials are essential;<br>• cannot distinguish variations among isotopes of an element;<br>• commercially available instruments are usually very limited in their ability to precisely and accurately measure the abundances of elements with $Z < 11$.
NAA (neutron activation analysis)	• non-destructive (except radiochemical NAA) and can measure many elements simultaneously;<br>• sample dissolution is not required before analysis (except radiochemical NAA);<br>• contamination due to acids and reagents can be avoided;<br>• samples can be in various forms (solids, liquids, gases and slurries);<br>• matrix effects often negligible;<br>• sample mass may vary from milligrams to several grams;<br>• high accuracy and precision;<br>• analysis is possible for nearly 75 individual elements (including certain organic elements at trace and ultra-trace concentrations);<br>• more than 30 elements can be simultaneously measured;<br>• high precision over long periods and excellent sensitivities.	• a nuclear reactor for activation is required;<br>• may poses safety concerns;<br>• several elements (e.g., Pb) can hardly be determined;<br>• generally useful for only specialised applications.
NMR (nuclear magnetic resonance)	• non-destructive and non-invasive nature;<br>• environmental friendly;<br>• relatively rapid and easy to use;<br>• minimum sample preparation;	• difficulties connected with overlapping of signals in multicomponent mixtures;<br>• information for major compounds may be enhanced, while that of minor components may be masked;

(Continued)

Table S1 *(Continued).*

Technique	Advantages	Drawbacks
NMR (nuclear magnetic resonance)	• short analysis time;<br>• quantitative and structural information for components of complex mixtures without pre-isolation;<br>• 2D NMR techniques can overcome the problems related to signals overlapping;<br>• repeatability and reproducibility of measurements over the long term;<br>• possibility to compare spectra originating from a single sample recorded by different spectrometers;<br>• long-term stability of spectra;<br>• no need for internal standardisation or calibration;<br>• small instrumental variability allows databases to be built;<br>• high-resolution NMR well suited in the profiling of biological materials.	• highly specialized operators are required;<br>• more expensive in comparison to other spectroscopic techniques.

[a)] Also known as graphite furnace atomic absorption spectroscopy (GFAAS).

[b)] Direct mercury analysis using the principle of in situ dry ashing followed by gold amalgamation and atomic absorption detection.

[c)] Also called ICP-atomic emission spectroscopy (ICP-AES).

[d)] Also called sector-field ICP-MS (SF-ICP-MS).

Table S2. Concentrations of the elements (means and standard deviations in brackets, concentrations expressed as mg kg^{-1}) in the reviewed studies.

Reference	Authentication issue	Product	Sample group	Measured elements and relative concentrations
		Fish		
[61]	Production method	Salmon	Wild	As, Ba, Be, Ca, Cd, Co, Cr, Cu, Fe, K, Mg, Mn, Na, Ni, P, Pb, Sr, Ti, Zn (not provided)
			Farmed	As, Ba, Be, Ca, Cd, Co, Cr, Cu, Fe, K, Mg, Mn, Na, Ni, P, Pb, Sr, Ti, Zn (not provided)
[33]	Geographical origin	Catfish	Auburn	Al (3.44 ± 2.69), Ca (1279 ± 580), Cr (0.28 ± 0.06), Cu (0.72 ± 0.08), Fe (10.60 ± 2.41), K (6121 ± 1142), Mg (1368 ± 51), Na (1557 ± 141), P (1,0462 ± 501), S (8230 ± 726), Zn (15.88 ± 2.37)
			Blackbelt	Al (7.14 ± 7.23), Ca (2852 ± 2571), Cr (0.57 ± 0.11), Cu (1.04 ± 0.47), Fe (8.04 ± 4.08), K (6253 ± 737), Mg (1090 ± 136), Na (1527 ± 314), P (9088 ± 1221), S (8263 ± 954), Zn (22.85 ± 3.89)
			Stoneville	Al (2.40 ± 2.06), Ca (426 ± 610), Cr (0.53 ± 0.24), Cu (0.64 ± 0.19), Fe (5.16 ± 1.44), K (8720 ± 2438), Mg (1139 ± 103), Na (1099 ± 125), P (9820 ± 793), S (9211 ± 836), Zn (19.37 ± 2.48)
[19]	Geographical origin	Croacker	Santos	As (13.59 ± 1.48), Br (26.72 ± 1.64), Ca (891.94 ± 109.72), Cd (0.00 ± 0.00), Cl (8073.50 ± 765.36), Cu (2.92 ± 0.06), Fe (24.41 ± 0.80), Hg (0.60 ± 0.10), K (18,886.00 ± 472.10), Pb (0.16 ± 0.03), Rb (2.42 ± 0.17), S (11,748.79 ± 537.25), Se (2.67 ± 0.32), Zn (19.87 ± 1.08)
			Parnaíba	As (24.15 ± 3.85), Br (25.38 ± 1.67), Ca (1437.75 ± 240.66), Cd (0.01 ± 0.01), Cl (6475.96 ± 635.00), Cu (5.53 ± 1.12), Fe (27.53 ± 1.32), Hg (0.09 ± 0.00), K (11,399.29 ± 240.66), Pb (0.15 ± 0.04), Rb (3.46 ± 0.52), S (11,895.54 ± 1478.63), Se (8.02 ± 1.85), Zn (18.49 ± 0.79)
	Seasonality		July	As (10.69 ± 0.39), Br (26.54 ± 2.66), Ca (536.63 ± 27.47), Cd (0.02 ± 0.00), Cl (4503.41 ± 521.02), Cu (3.42 ± 0.18), Fe (15.61 ± 1.37), Hg (0.84 ± 0.10), K (12,431.58 ± 664.64), Pb (0.27 ± 0.02), Rb (2.60 ± 0.20), S (8693.01 ± 334.37), Se (3.94 ± 1.04), Zn (18.72 ± 0.79)
			December	As (11.69 ± 0.18), Br (22.54 ± 0.71), Ca (715.28 ± 12.13), Cd (0.00 ± 0.00), Cl (4857.83 ± 1148.07), Cu (2.56 ± 0.26), Fe (15.41 ± 0.74), Hg (0.68 ± 0.01), K (17,856.86 ± 1539.46), Pb (0.00 ± 0.00), Rb (2.32 ± 0.17), S (11,299.06 ± 891.39), Se (3.18 ± 0.77), Zn (18.16 ± 1.15)
[48]	Geographical origin	European seabass	Croatia	As (0.60 ± 0.15), Ca (297.15± 0.39), Cd (0.01 ± 0.01), Co (0.02 ± 0.01), Cr (0.20 ± 0.05), Cu (0.88 ± 0.16), Fe (4.18 ± 0.76), Hg (0.17 ± 0.16), K (3675.85 ± 265.53), Mg (373.54 ± 67.66), Mn (0.20 ± 0.04), Na (545.33 ± 113.41), Ni (0.04 ± 0.02), P (3019.80 ± 229.14), Pb (0.20 ± 0.25), S (7856.48 ± 3146.50), Se (0.19 ± 0.07), Zn (6.39 ± 0.78)

(Continued)

Table S2. *(Continued).*

Reference	Authentication issue	Product	Sample group	Measured elements and relative concentrations
[48]	Geographical origin	European seabass	Greece	As (0.50 ± 0.14), Ca (130.99 ± 27.83), Cd (0.01 ± 0.01), Co (0.00 ± 0.00), Cr (0.22 ± 0.03), Cu (0.76 ± 0.13), Fe (3.50 ± 0.62), Hg (0.09 ± 0.08), K (3141.99 ± 257.58), Mg (321.97± 18.65), Mn (0.20 ± 0.05), Na (538.18 ± 125.79), Ni (0.03 ± 0.01), P (2616.39 ± 224.39), Pb (0.10 ± 0.12), S (6005.52 ± 2315.69), Se (0.22 ± 0.05), Zn (6.52 ± 0.37)
			Italy	As (0.56 ± 0.22), Ca (173.64 ± 110.05), Cd (0.01 ± 0.01), Co (0.01 ± 0.01), Cr (0.22 ± 0.03), Cu (0.72 ± 0.18), Fe (4.42 ± 0.91), Hg (0.06 ± 0.05), K (3412.42 ± 370.27), Mg (334.05 ± 49.77), Mn (0.19 ± 0.06), Na (544.27 ± 131.82), Ni (0.03 ± 0.01), P (2821.51 ± 260.28), Pb (0.08 ± 0.06), S (8608.63 ± 1875.33), Se (0.25 ± 0.08), Zn (6.29 ± 0.92)
			Turkey	As (0.64 ± 0.29), Ca (138.57 ± 43.24), Cd (0.01 ± 0.01), Co (0.01 ± 0.00), Cr (0.21 ± 0.01), Cu (0.77 ± 0.18), Fe (3.65 ± 0.99), Hg (0.06 ± 0.02), K (3195.65 ± 236.92), Mg (326.44 ± 19.69), Mn (0.22 ± 0.05), Na (559.24 ± 158.52), Ni (0.03 ± 0.01), P (2623.55 ± 194.36), Pb (0.07 ± 0.03), S (6778.13 ± 2007.43), Se (0.21 ± 0.05), Zn (6.07 ± 0.62)
	Production method		Wild	As (0.62 ± 0.42), Ca (157.31± 49.82), Cd (0.01 ± 0.00), Co (0.01 ± 0.01), Cr (0.22 ± 0.03), Cu (0.66 ± 0.19), Fe (4.20 ± 0.95), Hg (0.07 ± 0.07), K (3274.94 ± 264.28), Mg (326.96 ± 38.69), Mn (0.17 ± 0.04), Na (600.41 ± 134.13), Ni (0.03 ± 0.02), P (2669.40 ± 200.85), Pb (0.09 ± 0.07), S (7982.54 ± 2050.61), Se (0.28 ± 0.09), Zn (5.96 ± 0.76)
	Farming system		Extensively rared	As (0.62 ± 0.42), Ca (157.31± 49.82), Cd (0.01 ± 0.00), Co (0.01 ± 0.01), Cr (0.22 ± 0.03), Cu (0.66 ± 0.19), Fe (4.20 ± 0.95), Hg (0.07 ± 0.07), K (3274.94 ± 264.28), Mg (326.96 ± 38.69), Mn (0.17 ± 0.04), Na (600.41 ± 134.13), Ni (0.03 ± 0.02), P (2669.40 ± 200.85), Pb (0.09 ± 0.07), S (7982.54 ± 2050.61), Se (0.28 ± 0.09), Zn (5.96 ± 0.76)
			Semi-intensively rared	As (0.64 ± 0.27), Ca (158.31 ± 157.98), Cd (0.01 ± 0.01), Co (0.01 ± 0.00), Cr (0.22 ± 0.02), Cu (0.66 ± 0.07), Fe (4.22 ± 0.72), Hg (0.07 ± 0.04), K (3272.16 ± 386.70), Mg (332.91 ± 45.11), Mn (0.19 ± 0.12), Na (464.52 ± 123.28), Ni (0.03 ± 0.01), P (2712.32 ± 250.13), Pb (0.06 ± 0.02), S (6867.19 ± 2591.31), Se (0.26 ± 0.08), Zn (6.46 ± 1.28)
			Intensively rared	As (0.56 ± 0.18), Ca (183.98 ± 122.53), Cd (0.01 ± 0.01), Co (0.01 ± 0.01), Cr (0.22 ± 0.42), Cu (0.79 ± 0.18), Fe (4.20 ± 1.01), Hg (0.08 ± 0.08), K (3393.76 ± 372.46), Mg (338.54 ± 51.63), Mn (0.20 ± 0.04), Na (559.96 ± 131.40), Ni (0.03 ± 0.01), P (2811.33 ± 285.98), Pb (0.10 ± 0.13), S (7918.98 ± 2388.37), Se (0.22 ± 0.06), Zn (6.32 ± 0.72)

(Continued)

Table S2. *(Continued).*

Reference	Authentication issue	Product	Sample group	Measured elements and relative concentrations
[49]	Geographical origin	Asian seabass	Malaysia	Al, As, At, Bi, Br, Ca, Cd, Cl, Cr, Cu, Fe, Hf, K, Mg, Mn, Nd, Ni, P, Pb, Rb, S, Sb, Se, Si, Sn, Sr, Ti, U, Y, Zn, Zr (not provided)
			Northern site	Al, As, At, Bi, Br, Ca, Cd, Cl, Cr, Cu, Fe, Hf, K, Mg, Mn, Nd, Ni, P, Pb, Rb, S, Sb, Se, Si, Sn, Sr, Ti, U, Y, Zn, Zr (not provided)
			Queensland	Al, As, At, Bi, Br, Ca, Cd, Cl, Cr, Cu, Fe, Hf, K, Mg, Mn, Nd, Ni, P, Pb, Rb, S, Sb, Se, Si, Sn, Sr, Ti, U, Y, Zn, Zr (not provided)
	Production method		Wild	Al, As, At, Bi, Br, Ca, Cd, Cl, Cr, Cu, Fe, Hf, K, Mg, Mn, Nd, Ni, P, Pb, Rb, S, Sb, Se, Si, Sn, Sr, Ti, U, Y, Zn, Zr (not provided)
			Farmed	Al, As, At, Bi, Br, Ca, Cd, Cl, Cr, Cu, Fe, Hf, K, Mg, Mn, Nd, Ni, P, Pb, Rb, S, Sb, Se, Si, Sn, Sr, Ti, U, Y, Zn, Zr (not provided)
[62]	Geographical origin	European seabass*	Central Mediterannean	Er (0.38 ± 0.20), Eu (0.19 ± 0.50), Ho (0.09 ± 0.05), La (5.13 ± 3.18), Lu (0.05 ± 0.03), Tb (2.04 ± 1.51)
			West Mediterranean	Er (0.37 ± 0.26), Eu (0.34 ± 0.75), Ho (0.09 ± 0.06), La (5.01 ± 3.37), Lu (0.05 ± 0.04), Tb (2.09 ± 1.56)
			East Mediterranean	Er (0.29 ± 0.22), Eu (0.18 ± 0.54), Ho (0.07 ± 0.05), La (4.06 ± 3.36), Lu (0.04 ± 0.03), Tb (1.63 ± 1.50)
			Wild	Er (0.32 ± 0.26), Eu (0.16 ± 0.51), Ho (0.08 ± 0.05), La (4.61 ± 3.68), Lu (0.04 ± 0.03), Tb (1.80 ± 2.03)
			Farmed	Er (0.36 ± 0.22), Eu (0.27 ± 0.63), Ho (0.08 ± 0.05), La (4.92 ± 3.22), Lu (0.05 ± 0.03), Tb (2.03 ± 1.55)
		Echinoderms		
[23]	Geographical origin	Sea cucumber	Yellow Sea	Al, As, Cd, Co, Cr, Cu, Fe, Hg, Mn, Mo, Ni, Pb, Se, V, Zn (not provided)
			Bohai Sea	Al, As, Cd, Co, Cr, Cu, Fe, Hg, Mn, Mo, Ni, Pb, Se, V, Zn (not provided)
			East China Sea	Al, As, Cd, Co, Cr, Cu, Fe, Hg, Mn, Mo, Ni, Pb, Se, V, Zn (not provided)

(Continued)

Table S2. *(Continued).*

Reference	Authentication issue	Product	Sample group	Measured elements and relative concentrations
[63]	Geographical origin	Sea cucumber	Weihai	Ag (1.42 ± 0.35), Al (36.25 ± 31.81), As$^{\#}$ (1152.20 ± 158.29), Ba (0.29 ± 0.18), Bi (0.56 ± 0.21), Ca (953.20 ± 288.59), Cd$^{\#}$ (15.88 ± 8.18), Ce$^{\#}$ (78.59 ± 38.79), Co$^{\#}$ (28.95 ± 16.34), Cr$^{\#}$ (0.11 ± 0.07), Cu (0.24 ± 0.16), Dy$^{\#}$ (3.99 ± 2.56), Er$^{\#}$ (2.39 ± 1.16), Eu$^{\#}$ (1.23 ± 0.85), Fe$^{\#}$ (50.93 ± 37.76), Gd$^{\#}$ (7.90 ± 3.50), Ho$^{\#}$ (0.70 ± 0.46), K (934.10 ± 251.14), La$^{\#}$ (42.01 ± 20.81), Li (0.21 ± 0.08), Lu$^{\#}$ (0.26 ± 0.17), Mg (1320.67 ± 222.71), Mn (4.63 ± 3.38), Na (10181.32 ± 1557.81), Nd$^{\#}$ (33.51 ± 21.19), Ni$^{\#}$ (71.78 ± 42.33), Pb$^{\#}$ (28.04 ± 14.16), Pr$^{\#}$ (8.43 ± 4.87), Sc$^{\#}$ (19.22 ± 9.63), Se (0.28 ± 0.12), Sm$^{\#}$ (5.92 ± 4.07), Sn$^{\#}$ (7.41 ± 3.78), Sr (7.20 ± 1.58), Tb$^{\#}$ (0.77 ± 0.51), Tm$^{\#}$ (0.60 ± 1.34), V$^{\#}$ (1515.90 ± 199.49), Y$^{\#}$ (30.09 ± 17.19), Yb$^{\#}$ (1.67 ± 1.11), Zn (2.63 ± 0.95)
			Dalian	Ag (4.67 ± 5.41), Al (17.75 ± 13.99), As$^{\#}$ (1187.80 ± 101.780), Ba (0.24 ± 0.18), Bi (0.4 ± 0.19), Ca (1040.70 ± 125.21), Cd$^{\#}$ (12.85 ± 3.01), Ce$^{\#}$ (29.39 ± 35.97), Co$^{\#}$ (29.91 ± 4.99), Cr$^{\#}$ (0.11 ± 0.03), Cu (0.28 ± 0.02), Dy$^{\#}$ (1.48 ± 1.26), Er$^{\#}$ (0.67 ± 0.68), Eu$^{\#}$ (0.45 ± 0.37), Fe$^{\#}$ (19.46 ± 14.13), Gd$^{\#}$ (2.27 ± 2.11), Ho$^{\#}$ (0.28 ± 0.24), K (996.70 ± 116.25), La$^{\#}$ (14.89 ± 12.03), Li (0.16 ± 0.02), Lu$^{\#}$ (0.12 ± 0.10), Mg (1242.73 ± 70.18), Mn (3.64 ± 0.93), Na (9261.34 ± 726.21), Nd$^{\#}$ (11.15 ± 10.07), Ni$^{\#}$ (99.32 ± 47.51), Pb$^{\#}$ (22.94 ± 20.97), Pr$^{\#}$ (2.76 ± 2.47), Sc$^{\#}$ (11.23 ± 6.04), Se (0.38 ± 0.06), Sm$^{\#}$ (2.04 ± 1.78), Sn$^{\#}$ (10.49 ± 3.38), Sr (7.37 ± 0.51), Tb$^{\#}$ (0.27 ± 0.23), Tm$^{\#}$ (0.11 ± 0.09), V$^{\#}$ (1385.60 ± 224.58), Y$^{\#}$ (13.42 ± 9.74), Yb$^{\#}$ (0.72 ± 0.61), Zn (3.47 ± 0.59)
			Yingkou	Ag (2.24 ± 0.87), Al (15.66 ± 6.55), As$^{\#}$ (1345.40 ± 273.82), Ba (0.31 ± 0.11), Bi (1.67 ± 1.40), Ca (897.20 ± 387.47), Cd$^{\#}$ (78.66 ± 40.73), Ce$^{\#}$ (49.41 ± 15.17), Co$^{\#}$ (19.77 ± 5.93), Cr$^{\#}$ (0.09 ± 0.04), Cu (0.19 ± 0.13), Dy$^{\#}$ (2.62 ± 0.85), Er$^{\#}$ (4.01 ± 2.53), Eu$^{\#}$ (0.76 ± 0.30), Fe$^{\#}$ (20.45 ± 9.78), Gd$^{\#}$ (6.02 ± 2.07), Ho$^{\#}$ (0.47 ± 0.16), K (687.70 ± 251.80), La$^{\#}$ (28.48 ± 8.60), Li (0.16 ± 0.02), Lu$^{\#}$ (0.16 ± 0.07), Mg (1081.93 ± 283.14), Mn (1.87 ± 1.20), Na (7824.40 ± 2190.93), Nd$^{\#}$ (19.87 ± 7.33), Ni$^{\#}$ (72.88 ± 27.33), Pb$^{\#}$ (30.93 ± 11.25), Pr$^{\#}$ (5.40 ± 1.91), Sc$^{\#}$ (13.33 ± 4.24), Se (0.31 ± 0.14), Sm$^{\#}$ (3.24 ± 1.40), Sn$^{\#}$ (15.88 ± 5.88), Sr (8.23 ± 2.01), Tb$^{\#}$ (0.49 ± 0.18), Tm$^{\#}$ (0.16 ± 0.06), V$^{\#}$ (1957.10 ± 606.10), Y$^{\#}$ (23.82 ± 7.71), Yb$^{\#}$ (1.08 ± 0.46), Zn (2.95 ± 0.49)

(Continued)

Table S2. *(Continued).*

Reference	Authentication issue	Product	Sample group	Measured elements and relative concentrations
[63]	Geographical origin	Sea cucumber	Huludao	Ag (1.90 ± 0.64), Al (61.96 ± 43.69), As# (1248.70 ± 392.54), Ba (0.40 ± 0.26), Bi (0.78 ± 0.34), Ca (1105.50 ± 195.06), Cd# (56.60 ± 23.71), Ce# (122.13 ± 81.28), Co# (35.75 ± 9.39), Cr# (0.14 ± 0.05), Cu (0.38 ± 0.06), Dy# (6.72 ± 4.99), Er# (3.47 ± 2.61), Eu# (2.10 ± 1.47), Fe# (69.95 ± 56.70), Gd# (10.72 ± 7.52), Ho# (1.22 ± 0.88), K (810.50 ± 184.79), La# (62.54 ± 42.10), Li (0.22 ± 0.07), Lu# (0.45 ± 0.32), Mg (1257.42 ± 180.70), Mn (4.51 ± 3.36), Na (8651.48 ± 1650.74), Nd# (56.21 ± 39.15), Ni# (101.03 ± 38.72), Pb# (51.19 ± 30.28), Pr# (13.82 ± 9.40), Sc# (22.53 ± 16.80), Se (0.24 ± 0.08), Sm# (10.15 ± 7.19), Sn# (10.83 ± 4.86), Sr (7.80 ± 3.16), Tb# (1.27 ± 0.93), Tm# (0.45 ± 0.35), V# (1296.60 ± 220.85), Y# (52.02 ± 33.76), Yb# (2.93 ± 2.21), Zn (3.67 ± 0.83)
			Jinzhou	Ag (2.15 ± 0.38), Al (85.41 ± 52.14), As# (1454.70 ± 256.51), Ba (0.56 ± 0.21), Bi (0.80 ± 0.40), Ca (1397.30 ± 132.51), Cd# (121.60 ± 16.23), Ce# (200.65 ± 119.30), Co# (50.81 ± 6.27), Cr# (0.22 ± 0.03), Cu (0.42 ± 0.02), Dy# (11.32 ± 6.54), Er# (5.92 ± 3.44), Eu# (3.41 ± 1.96), Fe# (110.10 ± 68.77), Gd# (18.49 ± 10.85), Ho# (2.03 ± 1.17), K (995.20 ± 94.01), La# (103.34 ± 60.27), Li (0.24 ± 0.05), Lu# (0.74 ± 0.41), Mg (1549.90 ± 60.11), Mn (9.68 ± 5.53), Na (11591.34 ± 623.08), Nd# (98.06 ± 58.51), Ni# (145.05 ± 21.07), Pb# (46.73 ± 23.88), Pr# (23.65 ± 14.19), Sc# (36.45 ± 23.86), Se (0.20 ± 0.05), Sm# (17.93 ± 10.61), Sn# (6.00 ± 2.37), Sr (7.63 ± 0.42), Tb# (2.18 ± 1.27), Tm# (0.75 ± 0.42), V# (1353.30 ± 178.33), Y# (83.86 ± 43.53), Yb# (4.75 ± 2.78), Zn (3.41 ± 0.39)
		Crustaceans		
[64]	Geographical origin	Pacific white shrimp	Farm 1 (USA)	Al (65.2 ± 38.8), As (0.89 ± 0.20), Ba (2.32 ± 1.67), Ca (2351 ± 730), Co (0.05 ± 0.03), Cr (1.31 ± 0.87), Cu (28.8 ± 2.82), Fe (43.3 ± 29.9), K (10874 ± 1546), Mg (1119 ± 50.0), Mn (1.18 ± 0.56), Mo (0.08± 0.09), Na (4892 ± 320), Ni (0.42 ± 0.61), P (8989 ± 624), S (7657 ± 308), Se (1.62 ± 0.54), Ti (3.09 ± 2.25), Zn (49.5 ± 2.03), Zr (0.03 ± 0.03)
			Farm 2 (USA)	Al (134 ± 81.0), As (2.45 ± 0.94), Ba (0.32 ± 0.22), Ca (2039 ± 423), Co (0.03 ± 0.02), Cr (1.23 ± 0.53), Cu (26.3 ± 2.54), Fe (95.4 ± 53.4), K (11344 ± 1383), Mg (1474 ± 108), Mn (1.18 ± 0.67), Mo (0.03 ± 0.05), Na (4606 ± 238), Ni (0.35 ± 0.26), P (9845 ± 557), S (7391 ± 402), Se (2.00 ± 0.32), Ti (7.48 ± 4.06), Zn (50.5 ± 4.18), Zr (0.04 ± 0.04)

(Continued)

Table S2. *(Continued).*

Reference	Authentication issue	Product	Sample group	Measured elements and relative concentrations
[64]	Geographical origin	Pacific white shrimp	Farm 3 (USA)	Al (73.0 ± 62.8), As (2.2 ± 0.22), Ba (0.76 ± 0.35), Ca (2538 ± 993), Co (0.05 ± 0.03), Cr (0.83 ± 0.12), Cu (24.8 ± 5.07), Fe (41.1 ± 34.8), K (7938 ± 1393), Mg (1516 ± 259), Mn (1.71 ± 0.81), Mo (0.12 ± 0.10), Na (6488 ± 1613), Ni (0.1 ± 0.13), P (8503 ± 995), S (7481 ± 489), Se (1.84 ± 0.38), Ti (2.62 ± 2.65), Zn (48.7 ± 5.4), Zr (0.15 ± 0.10)
[65]	Geographical origin<br>Production method	Shrimps	FAO 71	As (77.64 ± 58.84), Cd (0.19 ± 0.20), P (12490 ± 264), Pb (0.04 ± 0.01), S (11778 ± 2302)
			Argentina	As (13.70 ± 4.61), Cd (0.11 ± 0.03), P (12664 ± 307), Pb (0.04 ± 0.01), S (13383 ± 1038)
			North Atlantic	As (17.42 ± –), Cd (0.05 ± –), P (9390 ± –), Pb (0.05 ± –), S (8456 ± –), S (14383 ± 138)
			Farm A	As (4.39 ± 0.27), Cd (0.02 ± 0.00), P (7401 ± 383), Pb (0.04 ± 0.00), S (7054 ± 271)
			Farm B	As (4.44 ± 1.35), Cd (0.01 ± 0.00), P (12404 ± 584), Pb (0.06 ± 0.00), S (9757 ± 730)
			Farm C	As (4.44 ± 1.35), Cd (0.01 ± 0.00), P (12404 ± 584), Pb (0.06 ± 0.00), S (9757 ± 730)
			Mozambique	As (57.14 ± 34.45), Cd (0.06 ± 0.03), P (12897 ± 759), Pb (0.04 ± 0.02), S (9392 ± 757)
			Nigeria	As (46.83 ± 24.67), Cd (0.04 ± 0.03), P (11600 ± 529), Pb (0.04 ± 0.02), S (10973 ± 764)
			Senegal	As (18.51 ± 3.20), Cd (0.06 ± 0.03), P (9807 ± 612), Pb (0.04 ± 0.01), S (11389 ± 822)
			Wild	As (4.41 ± 0.87), Cd (0.01 ± 0.01), P (9903 ± 2776), Pb (0.05 ± 0.01), S (8406 ± 1560)
			Farmed	As (44.75 ± 40.88), Cd (0.09 ± 0.11), P (11734 ± 1412), Pb (0.04 ± 0.01), S (11075 ± 1820)
			F. indicus	As (25.42 ± 4.56), Cd (0.02 ± 0.02), P (12889 ± 757), Pb (0.04 ± 0.01), S (10026 ± 346)
			F. merguiensis	As (77.64 ± 58.84), Cd (0.19 ± 0.20), P (12490 ± 264), Pb (0.04 ± 0.01), S (10404 ± 1053)
			F. notialis	As (27.95 ± 18.95), Cd (0.06 ± 0.03), P (10404 ± 1053), Pb (0.04 ± 0.01), S (11250 ± 1782)
			L. vannamei	As (4.41 ± 0.87), Cd (0.01 ± 0.01), P (9903 ± 2776), Pb (0.05 ± 0.01), S (8406 ± 1560)
			P. borealis	As (17.42 ± –), Cd (0.05 ± –), P (9390 ± –), Pb (0.05 ± –), S (8456 ± –)
			P. monodon	As (88.86 ± 10.09), Cd (0.07 ± 0.04), P (12904 ± 391), Pb (0.05 ± 0.03), S (8759 ± 333)
			P. muelleri	As (4.61 ± 9.19), Cd (0.11 ± 0.03), P (12664 ± 307), Pb (0.04 ± 0.01), S (13383 ± 1038)

(Continued)

Table S2. *(Continued).*

Reference	Authentication issue	Product	Sample group	Measured elements and relative concentrations
[66]	Geographical origin	Prawns	Australian	Al (37.31 ± 26.80), As (52.93 ± 45.82), B (3.52 ± 1.09), Cd (0.66 ± 0.64), Co (0.07 ± 0.04), Cr (0.17 ± 0.08), Cu (22.07 ± 7.10), Fe (39.20 ± 22.63), Hg (0.21 ± 0.17), K (11820.96 ± 3963.81), Li (0.14 ± 0.03), Mn (2.42 ± 2.31), Mo (0.06 ± 0.02), Ni (0.22 ± 0.10), Se (1.78 ± 0.40), Sr (30.39 ± 11.79), Ti (1.50 ± 1.22), V (0.14 ± 0.05), Zn (65.70 ± 11.05)
			Imported	Al (22.60 ± 15.27), As (28.18 ± 33.95), B (3.60 ± 2.78), Cd (0.40 ± 0.66), Co (0.04 ± 0.02), Cr (0.59 ± 0.64), Cu (11.58 ± 7.20), Fe (28.11 ± 15.01), Hg (0.09 ± 0.12), K (7556.71 ± 4851.99), Li (0.14 ± 0.07), Mn (1.43 ± 0.89), Mo (0.06 ± 0.04), Ni (0.38 ± 0.34), Se (1.48 ± 0.70), Sr (27.18 ± 19.17), Ti (1.24 ± 0.58), V (0.09 ± 0.05), Zn (54.22 ± 12.06)
[22]	Geographical origin	Pacific white shrimps	India	Al (55.85 ± 7.77), As (0.62 ± 0.05) , B (176.63 ± 1.46), Ba (1.26 ± 0.08), Ca (1016.6 ± 69.6), Cd (6.77 ± 0.54), Co (0.45 ± 0.01), Cr (1.57 ± 0.11), Cu (13.73 ± 0.59), Fe (51.10 ± 7.92), K (5440.2 ± 65.9), Mg (834.8 ± 18.3), Mn (2.79 ± 0.30), Na (4282.0 ± 151.7), Ni (1.43 ± 0.89), P (6903.3 ± 167.7), Pb (0.43 ± 0.04), S (6193.3 ± 100.4), Se (3.28 ± 0.09), Si (18.68 ± 0.64), Ti (1.62 ± 0.21), Zn (24.19 ± 0.52), Zr (0.59 ± 0.02)
			Vietnam	Al (113.90 ± 16.48), As (3.58 ± 0.31) , B (248.7 ± 4.78), Ba (1.64 ± 0.50), Ca (1059.5 ± 76.8), Cd (4.81 ± 0.58), Co (0.38 ± 0.03), Cr (2.19 ± 0.19), Cu (31.38 ± 1.35), Fe (99.46 ± 15.42), K (4292.9 ± 90.1), Mg (1288.8 ± 22.4), Mn (6.66 ± 1.73), Na (6323.5 ± 201.9), Ni (1.43 ± 0.89), P (10,635.1 ± 232.6), Pb (0.69 ± 0.11), S (8458.3 ± 70.5), Se (3.65 ± 0.13), Si (19.49 ± 2.61), Ti (1.68 ± 0.21), Zn (41.58 ± 0.85), Zr (0.44 ± 0.02)
			Thailand	Al (71.07 ± 10.88), As (1.59 ± 0.14) , B (220.32 ± 3.01), Ba (0.83 ± 0.04), Ca (888.1 ± 31.0), Cd (4.64 ± 0.34), Co (0.36 ± 0.01), Cr (2.60 ± 0.18), Cu (27.43 ± 1.08), Fe (51.26 ± 8.12), K (4888.0 ± 104.0), Mg (1066.9 ± 20.9), Mn (3.18 ± 0.39), Na (5044.0 ± 124.0), Ni (1.43 ± 0.89), P (9206.6 ± 173.7), Pb (0.63 ± 0.05), S (6993.8 ± 110.5), Se (3.00 ± 0.12), Si (12.84 ± 0.97), Ti (1.02 ± 0.14), Zn (35.40 ± 0.79), Zr (0.40 ± 0.02)
[67]	Geographical origin	Chinese mitten crab	Site 1 (China)	Al (39.7 ± 13.7), Ba (224 ± 18), Ca (100975 ± 9166), Cu (36.1 ± 14.3), K (4938 ± 273), Mg (9433 ± 1046), Mn (127 ± 77.9), Na (7486 ± 609), Sr (776 ± 71), Zn (63.1 ± 7.3)
			Site 2 (China)	Al (22.5 ± 7.3), Ba (632 ± 68), Ca (86594 ± 11563), Cu (5.5 ± 3.3), K (4045 ± 258), Mg (6501 ± 704), Mn (51.0 ± 20.1), Na (6030 ± 478), Sr (942 ± 46), Zn (82.6 ± 6.8)

(Continued)

Table S2. *(Continued).*

Reference	Authentication issue	Product	Sample group	Measured elements and relative concentrations
[67]	Geographical origin	Chinese mitten crab	Site 3 (China)	Al (11.9 ± 11.5), Ba (395 ± 101), Ca (83385 ± 11400), Cu (21.0 ± 9.6), K (3976 ± 475), Mg (6302 ± 1140), Mn (60.9 ± 60.8), Na (6311 ± 489), Sr (1148 ± 258), Zn (76.9 ± 16.3)
			Site 4 (China)	Al (18.8 ± 6.7), Ba (648 ± 42), Ca (115547 ± 12964), Cu (6.7 ± 4.6), K (4471 ± 371), Mg (8292 ± 804), Mn (47.4 ± 12.4), Na (8147 ± 676), Sr (1145 ± 77), Zn (93.6 ± 5.5)
			Site 5 (China)	Al (20.1 ± 6.3), Ba (359 ± 64), Ca (111013 ± 8026), Cu (19.9 ± 7.5), K (4910 ± 402), Mg (8093 ± 1057), Mn (165 ± 42.2), Na (7156 ± 576), Sr (1173 ± 134), Zn (65.3 ± 7.3)
			Site 6 (China)	Al (19.1 ± 5.3), Ba (609 ± 107), Ca (110298 ± 7614), Cu (12.9 ± 7.0), K (4594 ± 648), Mg (10239 ± 843), Mn (35.6 ± 7.0), Na (8540 ± 853), Sr (1991 ± 141), Zn (87.6 ± 9.5)
			Site 7 (China)	Al (23.2 ± 8.8), Ba (661 ± 63), Ca (102058 ± 5223), Cu (9.3 ± 3.6), K (4285 ± 433), Mg (8215 ± 826), Mn (70.8 ± 18.7), Na (7727 ± 640), Sr (1030 ± 102), Zn (97.9 ± 6.8)
			Site 8 (China)	Al (25.7 ± 11.0), Ba (463 ± 48), Ca (103868 ± 15570), Cu (23.6 ± 9.3), K (4497 ± 419), Mg (6076 ± 1111), Mn (75.6 ± 47.3), Na (6009 ± 535), Sr (1107 ± 117), Zn (79.9 ± 6.5)
[68]	Seawater vs. Freshwater	Pacific white shrimps	Freshwater	Ag (0.01 ± 0.01), Al (21.36 ± 17.92), As (3.31 ± 1.43), Ba (0.66 ± 0.56), Cd (0.04 ± 0.03), Ce (0.0298 ± 0.0239), Co (0.07 ± 0.05), Cr (0.75 ± 0.41), Cs (0.06 ± 0.04), Cu (24.77 ± 7.05), Dy (0.0024 ± 0.0019), Er (0.0054 ± 0.0056), Eu (0.0005 ± 0.0004), Fe (44.53 ± 40.29), Ga (0.10 ± 0.08), Gd (0.0025 ± 0.0021), Ho (0.0005 ± 0.0005), Li (0.13 ± 0.03), Lu (0.0002 ± 0.0002), Mn (1.97 ± 1.68), Nd (0.0122 ± 0.0116), Ni (1.68 ± 2.27), Pb (0.09 ± 0.04), Pr (0.0030 ± 0.0026), Rb (4.08 ± 0.68), Sm (0.0027 ± 0.0026), Sr (20.67 ± 8.15), Tb (0.0004 ± 0.0004), Th (0.0048 ± 0.0050), Tm (0.0002 ± 0.0002), U (0.0247 ± 0.0383), V (0.05 ± 0.04), Y (0.0135 ± 0.0132), Yb (0.0011 ± 0.0013), Zn (42.31 ± 3.65)
			Sewater	Ag (0.01 ± 0.01), Al (133.83 ± 135.10), As (1.17 ± 0.51), Ba (2.43 ± 1.01), Cd (0.04 ± 0.06), Ce (0.2496 ± 0.2462), Co (0.10 ± 0.08), Cr (1.27 ± 0.73), Cs (0.05 ± 0.03), Cu (22.39 ± 6.26), Dy (0.0138 ± 0.0145), Er (0.0087 ± 0.0070), Eu (0.0047 ± 0.0044), Fe (110.78 ± 92.80), Ga (0.24 ± 0.26), Gd (0.0196 ± 0.0208), Ho (0.0026 ± 0.0026), Li (0.13 ± 0.07), Lu (0.0009 ± 0.0007), Mn (4.59 ± 2.85), Nd (0.1075 ± 0.1091), Ni (1.66 ± 3.45), Pb (0.24 ± 0.16), Pr (0.0275 ± 0.0268), Rb (4.05 ± 1.86), Sm (0.0220 ± 0.0231), Sr (12.26 ± 4.81), Tb (0.0027 ± 0.0028), Th (0.0341 ± 0.0327), Tm (0.0009 ± 0.0009), U (0.0079 ± 0.0040), V (0.31 ± 0.31), Y (0.0786 ± 0.0701), Yb (0.0054 ± 0.0057), Zn (47.33 ± 10.22)

(Continued)

Table S2. *(Continued).*

Reference	Authentication issue	Product	Sample group	Measured elements and relative concentrations
[25]	Geographical origin	Black tiger prawn	China	Al, As, At, Bi, Br, Ca, Cd, Cl, Cr, Cu, Fe, Hf, K, Mg, Mn, Nd, Ni, P, Pb, Rb, S, Sb, Se, Si, Sn, Sr, Ti, U, Y, Zn, Zr (not provided)
			India	Al, As, At, Bi, Br, Ca, Cd, Cl, Cr, Cu, Fe, Hf, K, Mg, Mn, Nd, Ni, P, Pb, Rb, S, Sb, Se, Si, Sn, Sr, Ti, U, Y, Zn, Zr (not provided)
			Indonesia	Al, As, At, Bi, Br, Ca, Cd, Cl, Cr, Cu, Fe, Hf, K, Mg, Mn, Nd, Ni, P, Pb, Rb, S, Sb, Se, Si, Sn, Sr, Ti, U, Y, Zn, Zr (not provided)
			Malayssia	Al, As, At, Bi, Br, Ca, Cd, Cl, Cr, Cu, Fe, Hf, K, Mg, Mn, Nd, Ni, P, Pb, Rb, S, Sb, Se, Si, Sn, Sr, Ti, U, Y, Zn, Zr (not provided)
			New South Wales	Al, As, At, Bi, Br, Ca, Cd, Cl, Cr, Cu, Fe, Hf, K, Mg, Mn, Nd, Ni, P, Pb, Rb, S, Sb, Se, Si, Sn, Sr, Ti, U, Y, Zn, Zr (not provided)
			Queensland	Al, As, At, Bi, Br, Ca, Cd, Cl, Cr, Cu, Fe, Hf, K, Mg, Mn, Nd, Ni, P, Pb, Rb, S, Sb, Se, Si, Sn, Sr, Ti, U, Y, Zn, Zr (not provided)
			West Australia	Al, As, At, Bi, Br, Ca, Cd, Cl, Cr, Cu, Fe, Hf, K, Mg, Mn, Nd, Ni, P, Pb, Rb, S, Sb, Se, Si, Sn, Sr, Ti, U, Y, Zn, Zr (not provided)
	Production method		Wild	Al, As, At, Bi, Br, Ca, Cd, Cl, Cr, Cu, Fe, Hf, K, Mg, Mn, Nd, Ni, P, Pb, Rb, S, Sb, Se, Si, Sn, Sr, Ti, U, Y, Zn, Zr (not provided)
			Farmed	Al, As, At, Bi, Br, Ca, Cd, Cl, Cr, Cu, Fe, Hf, K, Mg, Mn, Nd, Ni, P, Pb, Rb, S, Sb, Se, Si, Sn, Sr, Ti, U, Y, Zn, Zr (not provided)
		Molluscs		
[24]	Geographical origin	Mussels	Vigo (Spain)	Ag (26.81 ± –), As (8.86 ± –), Ba (2.4 ± –), Cd (0.59 ± –), Ce (267.31 ± –), Co (0.19 ± –), Cr (0.47 ± –), Cu (4.48 ± –), Dy# (18.52 ± –), Er# (9.63 ± –), Eu# (6.06 ± –), Ga (0.24 ± –), Gd# (26.93 ± –), Ho# (3.33 ± –), La# (142.32 ± –), Lu# (2.41 ± –), Mn (4.69 ± –), Mo (0.69 ± –), Nb# (19.81 ± –), Nd# (122.22 ± –), Ni (0.66 ± –), Pb (1.79 ± –), Pr# (31.44 ± –), Rb (2.98 ± –), Sb# (13.66 ± –), Se (1.95 ± –), Sm# (26.89 ± –), Sn (0.33 ± –), Sr (41.61 ± –), Ta# (13.18 ± –), Te# (1.44 ± –), Th# (46.44 ± –), Tl# (6.17 ± –), Tm# (1.37 ± –), U# (140.6 ± –), V (2.53 ± –), Y# (87.77 ± –), Yb# (7.25 ± –), Zn (118.54 ± –), Zr (0.09 ± –)

(Continued)

Table S2. *(Continued).*

Reference	Authentication issue	Product	Sample group	Measured elements and relative concentrations
[24]	Geographical origin	Mussels	Pontevedra (Spain)	Ag (46.03 ± –), As (7.59 ± –), Ba (0.5 ± –), Cd (1.29 ± –), Ce[#] (99.1 ± –), Co (0.2 ± –), Cr (0.27 ± –), Cu (3.82 ± –), Dy[#] (6.30 ± –), Er[#] (3.08 ± –), Eu[#] (1.89± –), Ga (0.06 ± –), Gd[#] (9.91 ± –), Ho[#] (0.95 ± –), La[#] (54.51 ± –), Lu[#] (0.68 ± –), Mn (4.00 ± –), Mo (2.48 ± –), Nb[#] (28.93 ± –), Nd[#] (45.2 ± –), Ni (0.27 ± –), Pb (0.68 ± –), Pr[#] (11.52 ± –), Rb (3.17 ± –), Sb[#] (4.05 ± –), Se (2.25 ± –), Sm[#] (9.35 ± –), Sn (0.10 ± –), Sr (31.21 ± –), Ta[#] (<LOD), Te[#] (<LOD), Th[#] (15.01 ± –), Tl[#] (3.33 ± –), Tm[#] (<LOD), U[#] (75.95 ± –), V (1.42 ± –), Y[#] (31.17 ± –), Yb[#] (1.94 ± –), Zn (101.44 ± –), Zr (0.03 ± –)
			Arousa (Spain)	Ag (19.92± –), As (9.64 ± –), Ba (1.13 ± –), Cd (0.63 ± –), Ce[#] (247.88 ± –), Co (2.3 ± –), Cr (1.18 ± –), Cu (3.96 ± –), Dy[#] (17.54 ± –), Er[#] (9.23 ± –), Eu[#] (5.41 ± –), Ga (0.11 ± –), Gd[#] (29.95 ± –), Ho[#] (2.93 ± –), La[#] (135.05 ± –), Lu[#] (1.72 ± –), Mn (5.7 ± –), Mo (0.68 ± –), Nb[#] (20.68 ± –), Nd[#] (123.12 ± –), Ni (1.24 ± –), Pb (0.73 ± –), Pr[#] (29.99 ± –), Rb (3.96 ± –), Sb[#] (7.4 ± –), Se (2.69 ± –), Sm[#] (25.83 ± –), Sn (0.18 ± –), Sr (24.76 ± –), Ta[#] (<LOD), Te[#] (1.71 ± –), Th[#] (37.39 ± –), Tl[#] (1.79 ± –), Tm[#] (<LOD), U[#] (66.64 ± –), V (1.08 ± –), Y[#] (87.81 ± –), Yb[#] (5.79 ± –), Zn (143.43 ± –), Zr (0.08 ± –)
			Muros-Noia (Spain)	Ag (17.00 ± –), As (7.89 ± –), Ba (0.77 ± –), Cd (0.92 ± –), Ce[#] (143.7 ± –), Co (0.18 ± –), Cr (0.36 ± –), Cu (3.79 ± –), Dy[#] (10.83 ± –), Er[#] (6.17 ± –), Eu[#] (3.96 ± –), Ga (0.09 ± –), Gd[#] (17.85 ± –), Ho[#] (2.05 ± –), La[#] (76.24 ± –), Lu[#] (1.8 ± –), Mn (3.79 ± –), Mo (1.30 ± –), Nb[#] (<LOD), Nd[#] (71.18 ± –), Ni (0.55 ± –), Pb (0.59 ± –), Pr[#] (17.39 ± –), Rb (2.98 ± –), Sb[#] (7.12 ± –), Se (2.08 ± –), Sm[#] (15.68 ± –), Sn (0.06 ± –), Sr (27.67 ± –), Ta[#] (<LOD), Te[#] (<LOD), Th[#] (27.06 ± –), Tl[#] (5.30 ± –), Tm[#] (0.99 ± –), U[#] (70.94 ± –), V (1.06 ± –), Y[#] (50.32 ± –), Yb[#] (4.39 ± –), Zn (77.32 ± –), Zr (0.03 ± –)
			Ares-Betanzos (Spain)	Ag (20.89 ± –), As (6.54 ± –), Ba (0.91 ± –), Cd (0.26 ± –), Ce[#] (206.32 ± –), Co (0.16 ± –), Cr (0.43 ± –), Cu (3.75 ± –), Dy[#] (15.30 ± –), Er[#] (8.86 ± –), Eu[#] (5.17 ± –), Ga (0.09 ± –), Gd[#] (27.11 ± –), Ho[#] (2.87 ± –), La[#] (112.98 ± –), Lu[#] (<LOD), Mn (3.67 ± –), Mo (1.36 ± –), Nb[#] (15.86 ± –), Nd[#] (101.19 ± –), Ni (0.36 ± –), Pb (0.67 ± –), Pr[#] (26.64 ± –), Rb (3.40 ± –), Sb[#] (7.35 ± –), Se (1.87 ± –), Sm[#] (22.05 ± –), Sn (0.08 ± –), Sr (26.08 ± –), Ta[#] (<LOD), Te[#] (1.92 ± –), Th[#] (30.13 ± –), Tl[#] (3.77 ± –), Tm[#] (1.20 ± –), U[#] (46.29 ± –), V (1.24 ± –), Y[#] (73.30 ± –), Yb[#] (5.70 ± –), Zn (75.77 ± –), Zr (0.13 ± –)

(Continued)

Table S2. *(Continued).*

Reference	Authentication issue	Product	Sample group	Measured elements and relative concentrations
[69]	Geographical origin	Manila clams	Site 1 (China)	Al (816 ± 294), As (12.1 ± 3.6), Ba# (6225 ± 1679), Cd# (357 ± 98), Ce# (2053 ± 400), Co# (1498 ± 332), Cs# (99.0 ± 29.0), Cu# (8428 ± 847), Fe (878 ± 219), K (3793 ± 548), La# (1782 ± 426), Mg (2858 ± 482), Mn (31.6 ± 4.9), Mo# (444 ± 89), Na (9792 ± 2820), Pb# (1080 ± 284), Pd# (15.2 ± 1.0), Rb# (2050 ± 239), Sb# (26.1 ± 5.0), Se# (2292 ± 598), Sn# (279 ± 83), Sr# (26.2 ± 6.0), U# (239 ± 38), V# (1410 ± 346), Zn (83.1 ± 16.2)
			Site 2 (China)	Al (672 ± 281), As (14.4 ± 5.1), Ba# (2951 ± 1092), Cd# (519 ± 207), Ce# (1797 ± 674), Co# (1687 ± 747), Cs# (122 ± 38), Cu# (10108 ± 3121), Fe (936 ± 306), K (5519 ± 1648), La# (1098 ± 370), Mg (1881 ± 469), Mn (27.8 ± 8.4), Mo# (435 ± 149), Na (12249 ± 2492), Pb# (941 ± 278), Pd# (13.8 ± 1.2), Rb# (2690 ± 579), Sb# (29.0 ± 8.4), Se# (2107 ± 549), Sn# (47.8 ± 25.5), Sr# (25.4 ± 8.4), U# (129 ± 49), V# (1521 ± 578), Zn (65.8 ± 10.2)
			Site 3 (China)	Al (500 ± 148), As (14.2 ± 1.6), Ba# (3594 ± 781), Cd# (517 ± 132), Ce# (1491 ± 284), Co# (1278 ± 108), Cs# (70.8 ± 12.7), Cu# (6844 ± 911), Fe (663 ± 113), K (7082 ± 628), La# (1103 ± 236), Mg (2426 ± 285), Mn (22.0 ± 5.7), Mo# (413 ± 73), Na (11159 ± 1869), Pb# (975 ± 286), Pd# (14.3 ± 1.0), Rb# (2981 ± 244), Sb# (19.2 ± 5.0), Se# (2046 ± 341), Sn# (29.5 ± 18.4), Sr# (35.7 ± 6.2), U# (197 ± 26), V# (949 ± 247), Zn (84.6 ± 11.4)
[70]	Geographical origin	Cuttlefish (ink)	Site 1 (Italy)	As (0.11 ± 0.01), Ca (973 ± 88.1), Cd (0.014 ± 0.004), Co (0.016 ± 0.0009), Cr (0.22 ± 0.03), Cu (4.21 ± 0.14), Fe (14.44 ± 7.64), Hg (0.20 ± 0.01), K (323.5 ± 24.1), Mg (2472.4 ± 297.6), Mn (0.31 ± 0.02), Mo (0.054 ± 0.003), Na (3467 ± 233.9), Ni (0.267 ± 0.01), P (451.3 ± 36.8), Pb (0.39 ± 0.03), V (0.013 ± 0.01), Zn (0.028 ± 0.005)
			Site 2 (Italy)	As (0.90 ± 0.04), Ca (2187 ± 147.2), Cd (0.09 ± 0.006), Co (0.024 ± 0.002), Cr (0.58 ± 0.13), Cu (8.54 ± 0.52), Fe (23.63 ± 6.69), Hg (0.90 ± 0.08), K (320.4 ± 15.07), Mg (4579 ± 244.8), Mn (0.71 ± 0.02), Mo (0.068 ± 0.008), Na (4705.08 ± 281.2), Ni (0.86 ± 0.01), P (393.6 ± 23.6), Pb (1.90 ± 0.24), V (0.203 ± 0.01), Zn (0.023 ± 0.004)
			Site 3 (Italy)	As (0.25 ± 0.01), Ca (1025.5 ± 97.6), Cd (0.023 ± 0.003), Co (0.021 ± 0.001), Cr (0.39 ± 0.07), Cu (5.98 ± 1.66), Fe (16.94 ± 7.23), Hg (0.22 ± 0.05), K (288.3 ± 9.09), Mg (2383 ± 244.3), Mn (0.35 ± 0.03), Mo (0.025 ± 0.004), Na (2524.2 ± 349), Ni (0.34 ± 0.03), P (335.7 ± 35.5), Pb (0.18 ± 0.005), V (0.079 ± 0.003), Zn (0.067 ± 0.01)

(Continued)

Table S2. *(Continued).*

Reference	Authentication issue	Product	Sample group	Measured elements and relative concentrations
[70]	Geographical origin	Cuttlefish (ink)	Site 4 (Italy)	As (0.20 ± 0.02), Ca (4823 ± 614.9), Cd (0.018 ± 0.003), Co (0.020 ± 0.001), Cr (0.23 ± 0.03), Cu (9.36 ± 0.35), Fe (29.14 ± 4.67), Hg (0.19 ± 0.01), K (385 ± 23.4), Mg (6246 ± 452.1), Mn (0.86 ± 0.03), Mo (0.081 ± 0.01), Na (5358 ± 171), Ni (0.172 ± 0.03), P (467.54 ± 32.61), Pb (0.54 ± 0.23), V (0.063 ± 0.008), Zn (0.045 ± 0.009)
			Site 5 (Italy)	As (0.39 ± 0.09), Ca (3087 ± 316.6), Cd (0.012 ± 0.004), Co (0.043 ± 0.004), Cr (0.21 ± 0.009), Cu (11.09 ± 1.28), Fe (35.95 ± 5.53), Hg (0.62 ± 0.09), K (522.7 ± 45.2), Mg (5998 ± 499), Mn (1.35 ± 0.17), Mo (0.12 ± 0.02), Na (6426 ± 531.7), Ni (0.215 ± 0.01), P (647.03 ± 34.6), Pb (0.28 ± 0.009), V (0.051 ± 0.005), Zn (0.068 ± 0.004)
			Site 6 (Italy)	As (0.24 ± 0.02), Ca (1300 ± 423.1), Cd (0.009 ± 0.001), Co (0.054 ± 0.008), Cr (0.17 ± 0.03), Cu (5.58 ± 0.31), Fe (26.42 ± 2.69), Hg (0.37 ± 0.09), K (259.7 ± 37.8), Mg (3714 ± 64.82), Mn (0.46 ± 0.18), Mo (0.034 ± 0.007), Na (3049 ± 519.6), Ni (0.316 ± 0.03), P (444.05 ± 17.59), Pb (0.29 ± 0.02), V (0.046 ± 0.008), Zn (0.096 ± 0.01)

#: Concentrations expressed as μg kg^{-1}

*: Concentrations expressed as ng g^{-1}
